# Transcriptome-wide association study identifies new susceptibility genes and pathways for depression

**DOI:** 10.1038/s41398-021-01411-w

**Published:** 2021-05-21

**Authors:** Xiaoyan Li, Xi Su, Jiewei Liu, Huijuan Li, Ming Li, the 23andMe Research Team, Wenqiang Li, Xiong-Jian Luo

**Affiliations:** 1 Key Laboratory of Animal Models and Human Disease Mechanisms of the Chinese Academy of Sciences & Yunnan Province, Kunming Institute of Zoology, Chinese Academy of Sciences, 650204 Kunming, Yunnan China; 2 Key Laboratory of Intelligent Computing and Signal Processing of Ministry of Education, Institutes of Physical Science and Information Technology, Anhui University, 230601 Hefei, Anhui China; 3 Henan Mental Hospital, The Second Affiliated Hospital of Xinxiang Medical University, Xinxiang, Henan China; 4 Henan Key Lab of Biological Psychiatry, International Joint Research Laboratory for Psychiatry and Neuroscience of Henan, Xinxiang Medical University, Xinxiang, Henan China; 5 KIZ-CUHK Joint Laboratory of Bioresources and Molecular Research in Common Diseases, Kunming Institute of Zoology, Chinese Academy of Sciences, 650204 Kunming, Yunnan China; 6 Center for Excellence in Animal Evolution and Genetics, Chinese Academy of Sciences, 650204 Kunming, China

**Keywords:** Genetics, Depression

## Abstract

Depression is the most prevalent mental disorder with substantial morbidity and mortality. Although genome-wide association studies (GWASs) have identified multiple risk variants for depression, due to the complicated gene regulatory mechanisms and complexity of linkage disequilibrium (LD), the biological mechanisms by which the risk variants exert their effects on depression remain largely unknown. Here, we perform a transcriptome-wide association study (TWAS) of depression by integrating GWAS summary statistics from 807,553 individuals (246,363 depression cases and 561,190 controls) and summary-level gene-expression data (from the dorsolateral prefrontal cortex (DLPFC) of 1003 individuals). We identified 53 transcriptome-wide significant (TWS) risk genes for depression, of which 23 genes were not implicated in risk loci of the original GWAS. Seven out of 53 risk genes (*B3GALTL*, *FADS1*, *TCTEX1D1*, *XPNPEP3*, *ZMAT2*, *ZNF501* and *ZNF502*) showed TWS associations with depression in two independent brain expression quantitative loci (eQTL) datasets, suggesting that these genes may represent promising candidates. We further conducted conditional analyses and identified the potential risk genes that driven the TWAS association signal in each locus. Finally, pathway enrichment analysis revealed biologically pathways relevant to depression. Our study identified new depression risk genes whose expression dysregulation may play a role in depression. More importantly, we translated the GWAS associations into risk genes and relevant pathways. Further mechanistic study and functional characterization of the TWS depression risk genes will facilitate the diagnostics and therapeutics for depression.

## Introduction

Depression is a complex and heterogeneous mental disorder characterized by depressed mood, loss of interests, appetite and sleep disturbances, cognitive impairments, feelings of worthlessness and hopelessness^1,2^. Depression has a high global prevalence (~4.7%) and over 298 million of the global population were affected by depression^3–5^. Females were more likely (about twice) to develop depressive symptoms than males^6^. As depression has a high prevalence and is accompanied with substantial morbidity and mortality, it becomes a leading cause of disability worldwide and a major contributor to global disease burden (e.g. the economic burden of depression was estimated about $210.5 billion in US in 2010^4,7^). To date, the etiology of depression remains largely unknown. However, accumulating evidence indicate that depression was caused by a combination of genetic and environmental factors. Twin study has estimated the heritability of depression to be ~37%^8^, indicating the important role of genetic component in the development of depression.

In the past decade, we have witnessed the rapid progress of genetic study of depression. Since CONVERGE consortium identified two genome-wide significant risk loci for depression in 2014^9^, multiple exciting findings have been reported by genome-wide association studies (GWASs)^10–13^. Recently, Howard et al. reported the largest GWAS of depression and identified over 100 risk loci that reached genome-wide significance level^14^. Despite the fact that GWASs of depression have made great progress in recent years and over 100 depression risk loci have been identified by GWASs, mechanistic investigations and biological interpretations lag far behind. For most of the risk loci, the implicated genes and corresponding mechanisms remain largely unknown. In traditional GWASs, the gene (or genes) that located the nearest to the most significant risk variant was usually assigned as the potential candidate gene. However, due to the complexity of LD (each risk locus usually contains several genes that were in high linkage disequilibrium (LD)) and gene regulation (genetic variants may regulate distal genes rather than the nearest gene through affecting chromosomal conformation change), the nearest gene may not necessarily represent the causal gene by which the identified GWAS risk variants exert their effects on depression^15^. As the vast majority of depression risk variants identified by GWASs were located in non-coding regions, it is possible that these risk variants confer depression risk through modulating gene expression rather than altering protein sequences or structures^16^.

Transcriptome-wide association study (TWAS) is a powerful approach aimed at identifying risk genes whose expression perturbations may confer disease susceptibility. Through integrating expression quantitative loci (eQTL) results with GWAS associations, TWAS could identify genes whose genetically regulated expression may be associated with diseases risk^17^. This method leverages a relatively small set of reference individuals with expression and genotype measured data to impute the expression-trait association statistics from GWAS summary data^18^. In addition to prioritizing putative target genes at genome-wide significant loci, TWAS provides an opportunity to detect genes with small effect sizes and located in regions that do not contain genome-wide significant variants^19^. Furthermore, the susceptibility genes identified by TWAS can more accurately inform follow-up experimental validation and potential treatment strategies.

In this study, we carried out a TWAS of depression by integrating brain eQTL data (from the dorsolateral prefrontal cortex (DLPFC)) of 1003 subjects (including the CommonMind Consortium (CMC)^20^ and the second phase of the BrainSeq Consortium (BrainSeq2)^21^) and the largest depression GWAS (including 246,363 cases and 561,190 controls)^14^. We identified 53 transcriptome-wide significant (TWS) depression risk genes that reached Bonferroni-corrected significance level. We subsequently performed conditional analysis of all significant TWAS associations to identify independent associations (i.e. the driven genes at each risk locus). Pathway and gene ontology analyses of the TWS genes identified relevant pathways, including formation of fibrin clot, female pregnancy and peripheral axonal degeneration pathways. Finally, we compared the expression level of the TWS depression risk genes in brains of depression cases and controls using RNA-sequencing (RNA-seq) expression data. Overall, our findings highlight the power of TWAS in identifying depression risk genes with small effect size and provide testable targets for further functional validation of depression. Future mechanistic investigations and functional experiments of the TWS depression risk genes will provide pivotal information for the diagnostics and therapeutics of depression.

## Methods and materials

### GWAS meta-analysis

The summary statistics from the largest GWAS of depression^14^ were used in this study. To identify the common risk variants for depression, Howard et al. performed a genome-wide meta-analysis through combining three large-scale depression cohorts (including 23andme, PGC2 and UK Biobank)^10,11,22^ (a total of 246,363 cases and 561,190 controls, after excluding overlapping samples) and identified 102 independent genetic variants associated with depression^14^. Detailed information about the participants ascertainment, genotyping, quality control and statistical analysis can be found in the original study^14^. The summary statistics for the meta-analysis of depression in UK Biobank and PGC_139k cohorts were downloaded from the Edinburgh data share center (10.7488/ds/2458)^14^. We then conducted a meta-analysis by combining summary statistics from above two cohorts and 23andMe^10^ using PLINK^23^ software and more detailed procedure of fixed-effect meta-analysis has been previously described in study of Li et al.^12^. We obtained the depression GWAS summary statistics of 23andme under a data transfer agreement^12^.

### Transcriptome-wide association analysis

We performed a TWAS through integrating the depression GWAS summary statistics and two sets of eQTL data (i.e. CMC^20^ (*N* = 452) and BrainSeq2^21^ (*N* = 551), respectively). The CMC SNP-expression weights (The SNP-expression weights represent the correlations between SNPs and gene expression in the reference panel while accounting for LD and were computed from different linear models (including BLUP, BSLMM, LASSO, Elastic Net and top SNPs)) were downloaded directly from the FUSION/TWAS website (http://gusevlab.org/projects/fusion/). The BrainSeq2 SNP-expression weights were obtained from the Lieber Institute for Brain Development (LIBD) browser (http://eqtl.brainseq.org/phase2/). Please refer to the original papers for further details on the sample collection, RNA extraction and sequencing, genotyping and statistical analysis^20,21^. The SNP-expression weights (i.e. expression weights) were derived using the default processing pipelines described in FUSION (http://gusevlab.org/projects/fusion)^17^.

The TWAS was performed using the FUSION software with default settings^17^ and a strict Bonferroni-corrected study-wise *P* threshold (i.e. *P* = 3.95 × 10^−6^ (0.05/12,647) (total number of genes across panels) was used in this study. TWAS analysis utilizes several regularized linear models (including BLUP, LASSO and elastic net) and an additional Bayesian linear mixed model (BSLMM) to evaluate expression imputation. Furthermore, FUSION performs a fivefold cross-validation for each of the desired models to determine which model is the best. For a given gene, SNP-expression weights in the cis-locus were computed using the best prediction model and FUSION typically restricts the cis-locus to 500 kb boundary on either side of the gene. The TWAS calculated Z-score results were used to assess the association between gene and depression and the absolute value of the Z-score reflects the association strength between implicated genes and disease. More detailed information about the principle of FUSION, statistical model and Z-score calculation can be found in the original paper^17^.

### Transcriptome-wide association analysis using SNP-expression weights from PsychENCODE

We further performed a TWAS using the SNP-expression weights data from PsychENCODE^24^. Briefly, TWAS was performed using the FUSION package^17^ (https://github.com/gusevlab/fusion_twas) as above described, with the use of SNP-expression weights from PsychENCODE (1321 unique individuals). Detailed information about PsychENCODE was provided in PsychENCODE website (http://resource.psychencode.org/) and related publication^17^.

### Transcriptome-wide association study in the Asian dataset

To further explore the ethnic difference for the TWAS results in depression across populations, we carried out a depression TWAS in the Asian population by integrating eQTL data from lymphoblastoid cell lines of 162 samples (including 80 Han Chinese from Beijing and 82 Japanese in Tokyo, Japan populations)^25^ and the Chinese GWAS summary statistics (including 5303 women depression and 5337 controls) from the CONVERGE consortium^9^. More detailed information about eQTL and GWAS datasets can be found in the original studies^9,25^.

### Defining of genes implicated in depression GWAS

We first extracted the genomic coordinates of all TWS genes (based on hg19). We then compared the genomic locations of the TWS genes with genes identified by Howard et al.^14^ (i.e. gene located in the 102 depression risk loci identified by Howard et al.). Genes that overlap with any risk locus defined by Howard et al. were considered as genes implicated in depression GWAS.

### Conditional and joint analysis

Conditional and joint analysis (based on genes rather than SNPs) were performed for genome-wide significant (Bonferroni-corrected) TWAS signals using FUSION^17^. The joint and conditional tests aim to evaluate the GWAS association signal after removing expression association from TWAS (i.e., to investigate if the GWAS signals are still significant after removing the expression association from TWAS). To evaluate the joint/conditional gene model, marginal association statistics (i.e., the main TWAS results) and a correlation/LD matrix are required. Each depression GWAS SNP association was conditioned on the joint gene model (one SNP at a time). The permutation test was used in conditional and joint analysis, with a maximum of 100,000 permutations and an initiate permutation *P*-value threshold of 0.05 for each feature. “FUSION.post_process.R” script was used for post-processing and generating multiple conditional output plots along with summary statistics.

### Pathway analysis

Considering the correlation (i.e., co-expression) of the TWAS implicated genes may affect the independence of the Bonferroni-correction assumption, we tend to relax the Bonferroni-corrected *P* threshold instead of stringent Bonferroni-corrected standard. Therefore, a relaxed Bonferroni significance threshold (estimated as *P* = 7.91 × 10^−6^ (0.10/12,647)) was used for pathway analyses in our analysis (which allows for more genes to include in the pathway enrichment analysis). GeneNetwork v2.0 (https://genenetwork.nl), which uses gene co-regulation to predict pathway membership and HPO term associations^26^ was used for pathway analysis. By integrating 31,499 public RNA-seq samples from a wide range of tissues and cell types, GeneNetwork generated multiple pathways for pathway analysis^26^. As genes are known to cause a particular disease or disease symptom tend to have similar molecular functions or are involved in the same pathway or biological processes^27,28^, it’s possible for GeneNetwork to accurately predict gene functions and to prioritize candidate disease genes with high accuracy. Agnostic analyses of pathways in databases (including Gene Ontology (GO) and Reactome) were done to identify special pathways relevant to depression. More detailed information about the principle of GeneNetwork such as principal component analysis (PCA), co-regulation scores calculation, *P*-value calculation and statistical analyses can be found in the original study^26^.

### Expression analysis of TWAS significant genes in depression cases and controls

TWAS identified a total of 53 genes that showed significant association with depression (after correcting for Bonferroni test). To explore whether the expression levels of depression-associated risk genes identified by TWAS were dysregulated in depression cases compared with controls, we obtained expression data (based on RNA-seq) of brain tissues (only the expression data from the DLPFC were used for further analysis) from three datasets, including GSE102556 (26 depression cases and 22 controls)^29^, GSE101521 (30 depression cases and 29 controls)^30^ and GSE80655 (23 depression cases and 24 controls)^31^. The same processing procedure was conducted for quality control, alignment and gene-expression quantification in three RNA-seq datasets. Briefly, Trimmomatic^32^ was utilized to examine the sequencing quality and trim reads. The clean paired-end reads were then aligned to the human reference genome (GRCh38) by using Hisat2^33^ and gene-level reads counts were quantified as transcripts per million (TPM) with featureCounts^34^. Protein-coding genes with average TPM ≥ 1.0 were extracted and differentially expressed genes (DEGs) were identified (based on read counts using likelihood ratio test (LRT)) for each of three datasets using DESeq2^35^ R package. More detailed information about sample collection, sample diagnose and RNA sequencing can be found in the original studies^36^.

### Spatio-temporal expression pattern analysis

To explore the spatio-temporal expression pattern of the depression candidate genes identified by TWAS analysis in human brain, we downloaded expression data (based on RNA-seq) from the Allen Institute for Brain Science (http://www.brainspan.org/)^37^. For a specific brain developmental stage, the mean expression level of all the genes in a geneset was represented as the expression level of the geneset at this stage. The gene-expression level was measured by RPKM (read per kilobase per million mapped reads) and two genesets implicated by TWAS analysis were used in this study. Background genes were extracted from a previous study^38^.

### Cell-type-specific expression analysis of TWS depression genes

The cell-type-specific expression pattern data were obtained from a previous study^39^. Briefly, Skene et al. carried out a comprehensive single-cell analysis of mouse brain tissues (including the hippocampus, neocortex, striatum and etc). A total of 9970 cells were sequenced and 24 cell types were identified by Skene et al. Skene et al. then calculated the specificity score of each gene in each cell type (a higher specificity score indicates a higher specificity of gene expression in a specific cell type). We firstly converted candidate human genes into mouse orthologs. We then extracted the specificity score for each gene in each cell type. We set a cutoff value of the specificity score to 0.1, and we calculated the number of genes with a specificity score greater than 0.1 in each cell type. All the analyses were performed with R software and more details about the single-cell data could be found in the original paper^39^.

### Spatio-temporal expression pattern analysis of TWS genes in the human brain

We used the expression data from the Allen Institute for Brain Science (http://www.brainspan.org/)^37^ for spatio-temporal expression analysis. Gene-expression values of the TWS risk genes in the prefrontal cortex (PFC) (*N* = 42) were downloaded and transformed as previously described^40^.

### Tissue-type-specific expression analysis of TWS depression genes

To explore the expression pattern of TWS genes in human tissues, we investigated their expression level in diverse human tissues using the Genotype-Tissue Expression (GTEx) project (http://gtexportal.org/)^41^, which includes expression data in 54 human tissues. Detailed information about the GTEx (e.g. sample source or size, gene-expression normalization) can be found in original publication^41^ and the GTEx website.

## Results

### TWAS identifies 53 susceptibility genes for depression

**Fig. 1:**
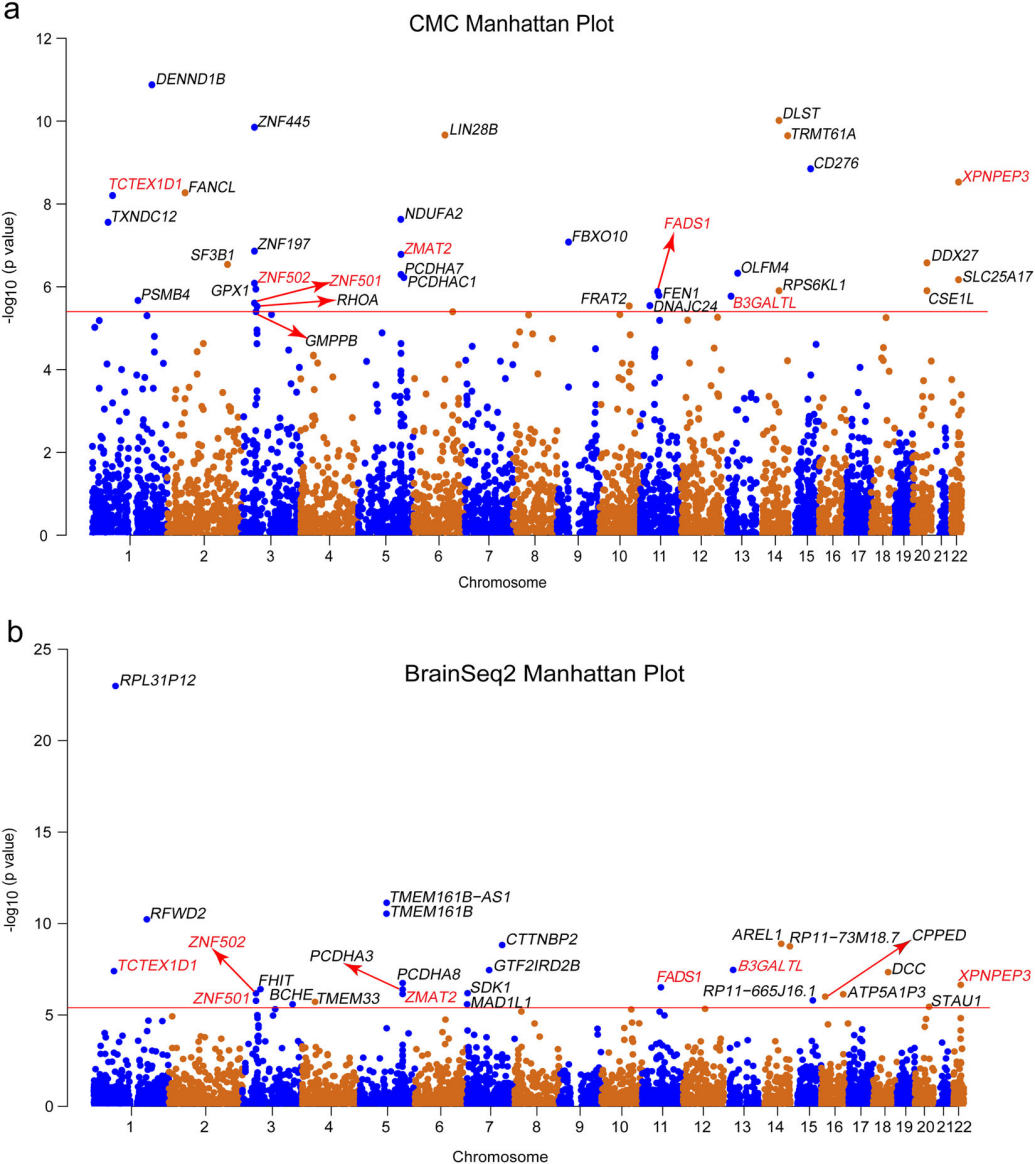
Manhattan plot of the TWAS results for depression (246,363 cases and 561,190 controls). **a** Manhattan plot of TWAS results in CMC dataset (a total of 33 significant genes detected). **b** Manhattan plot of TWAS results in BrainSeq2 dataset (a total of 27 significant genes detected). Each point represents a gene, with physical genomic position (chromosome, basepair) plotted on the *x*-axis and association *P-*value (the −log_10_ (FUSION *P*-value)) between gene expression in the DLPFC and depression plotted on the *y*-axis. Bonferroni-corrected significant genes are labeled and the significance threshold of *P* = 7.91 × 10^−6^ was used. Seven genes reached transcriptome-wide significance in both two expression panels (including *B3GALTL*, *FADS1*, *TCTEX1D1*, *XPNPEP3*, *ZMAT2*, *ZNF501* and *ZNF502*) and are highlighted in red colour.

To identify genes associated with depression, we performed a TWAS by integrating two gene-expression reference panels (i.e. CMC and BrainSeq2) and summary-level association data from the largest depression GWAS meta-analysis so far (a total of 807,553 individuals). We performed TWAS using the FUSION software (Methods)^17^. Briefly, this approach uses GWAS summary statistics and gene-expression panels with reference LD to estimate the association between the cis-genetic components of gene expression and depression risk. In total, we identified 53 TWS depression-associated genes (summed across two expression reference panels) after Bonferroni correction, including 33 significant genes detected using the CMC dataset (Supplementary Table 1 and Fig. 1a) and 27 genes detected using the BrainSeq2-DLPFC dataset (Supplementary Table 2 and Fig. 1b). Among the TWS associations (53 genes), 30 of the genes were implicated in the original depression GWAS and the remaining 23 genes did not fall within previous GWAS loci (definition of genes implicated in depression GWAS can be found in methods). Notably, we found that 7 out of 53 genes reached transcriptome-wide significance level in both two expression panels (CMC and BrainSeq2), including *B3GALTL*, *FADS1*, *TCTEX1D1*, *XPNPEP3*, *ZMAT2*, *ZNF501* and *ZNF502* (Table 1 and Fig. 1). Among the seven TWS genes, upregulation of *XPNPEP3* may be associated with depression risk as it has a positive Z-score (Z > 0 suggests that the gene is predicted to be upregulated in cases compared with controls). However, downregulation of other genes may increase risk of depression (Z < 0). These data suggested that the TWAS significant genes may have a role in depression risk. In addition, the overlapping genes identified in both expression panels represent plausible candidate genes for depression as these genes showed TWS association with depression in two independent eQTL datasets.

**Table 1:** Significant TWAS genes both in CMC and LIBD datasets for depression.

Gene	Region	CMC dataset			BrainSeq2 dataset		
		Best eQTL	TWAS.Z	TWAS.P	Best eQTL	TWAS.Z	TWAS.P
*TCTEX1D1*	chr 1:67218142–67244470	rs512691	−5.81	6.26E-09	rs10493416	−5.49	3.99E-08
*ZNF501*	chr 3:44771088–44778575	rs10514710	−4.69	2.68E-06	rs10514710	−4.79	1.68E-06
*ZNF502*	chr 3:44754135–44765323	rs10514710	−4.93	8.21E-07	rs10514710	−4.98	6.50E-07
*ZMAT2*^a^	chr 5:140078265–140086248	rs801183	−5.24	1.65E-07	rs3756341	−5.02	5.28E-07
*FADS1*^a^	chr 11:61567099–61596790	rs174568	−4.82	1.42E-06	rs174566	−5.12	3.08E-07
*B3GALTL*	chr 13:31774073–31906413	rs4065552	−4.79	1.70E-06	rs9543390	−5.52	3.44E-08
*XPNPEP3*	chr 22:41253081–41363838	rs138354	5.93	2.97E-09	rs2899341	5.18	2.26E-07

^a^Indicates that the TWAS gene was not implicated in the original depression GWAS.

### Expression signals driven the depression TWAS hits

**Fig. 2:**
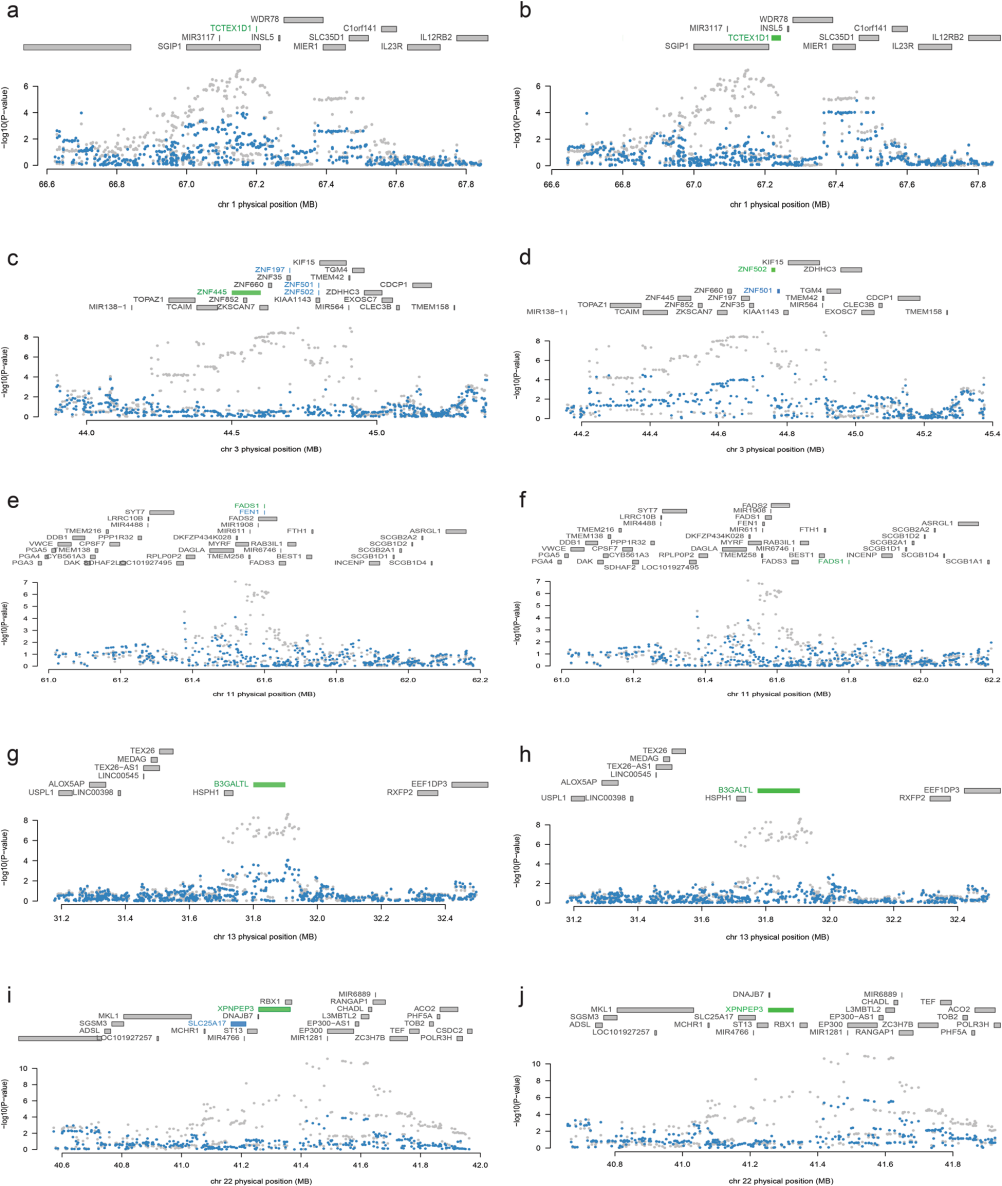
Regional association of transcriptome-wide significant genes. **a** Chr 1 regional association plot in CMC dataset. **b** Chr 1 regional association plot in BrainSeq2 dataset. Of note, *TCTEX1D1* driven the association signal in both two eQTL datasets. **c** Chr 3 regional association plot in CMC dataset. **d** Chr 3 regional association plot in BrainSeq2 dataset. **e** Chr 11 regional association plot in CMC dataset. **f** Chr 11 regional association plot in BrainSeq2 dataset. Notably, *FADS1* explained most of the association signal in both two eQTL datasets. **g** Chr 13 regional association plot in CMC dataset. **h** Chr 13 regional association plot in BrainSeq2 dataset. Notably, *B3GALTL* driven the association signal in both two eQTL datasets. **i** Chr 22 regional association plot in CMC dataset. **j** Chr 22 regional association plot in BrainSeq2 dataset. Notably, *XPNPEP3* explained most of the association signal in both two eQTL datasets. The top panel in each plot shows all of the genes in the locus. The marginally TWAS associated genes are highlighted in blue, and those that are jointly significant highlighted in green. The bottom panel shows a Manhattan plot of the GWAS data before (grey) and after (blue) conditioning on the predicted expression of the green genes. The *x*-axis denotes genome coordinates and the *y*-axis denotes association *P*-values in GWAS.

TWAS usually identifies several TWS genes for each of the risk locus. To detect if the identified transcriptome-wide association signals were conditionally independent and to explore whether the GWAS signals remain significant after removing the expression weights from TWAS, we conducted conditional and joint analyses. Our conditional analyses identified several independent TWS genes from both of the brain eQTL datasets (Fig. 2). We found that *TCTEX1D1* explains most of the signal at its locus in both CMC dataset (rs10789214 lead SNP *P*_GWAS_ = 7.53 × 10^−8^, conditioned on *TCTEX1D1* lead SNP *P*_GWAS_ = 7.83 × 10^−3^) (Fig. 2a) and BrainSeq2 dataset (rs10789214 lead SNP *P*_GWAS_ = 7.53 × 10^−8^, conditioned on *TCTEX1D1* lead SNP *P*_GWAS_ = 2.12 × 10^−2^) (Fig. 2b). Similarly, conditional analyses showed that *ZNF445* explained most of the signal at its locus in CMC dataset (Fig. 2c) and *ZNF502* explained most of the signal at its locus in BrainSeq2 dataset (Fig. 2d). In addition, we also found that *FADS1* (Fig. 2e, f), *B3GALTL* (Fig. 2g, h) and *XPNPEP3* (Fig. 2i, j) genes explained most of the variance at their loci in both CMC and BrainSeq2 datasets. Collectively, our conditional analyses identified independent genes that driven the transcriptome-wide association signals.

### Pathway enrichment analysis of the identified TWS genes revealed related biological processes

**Fig. 3:**
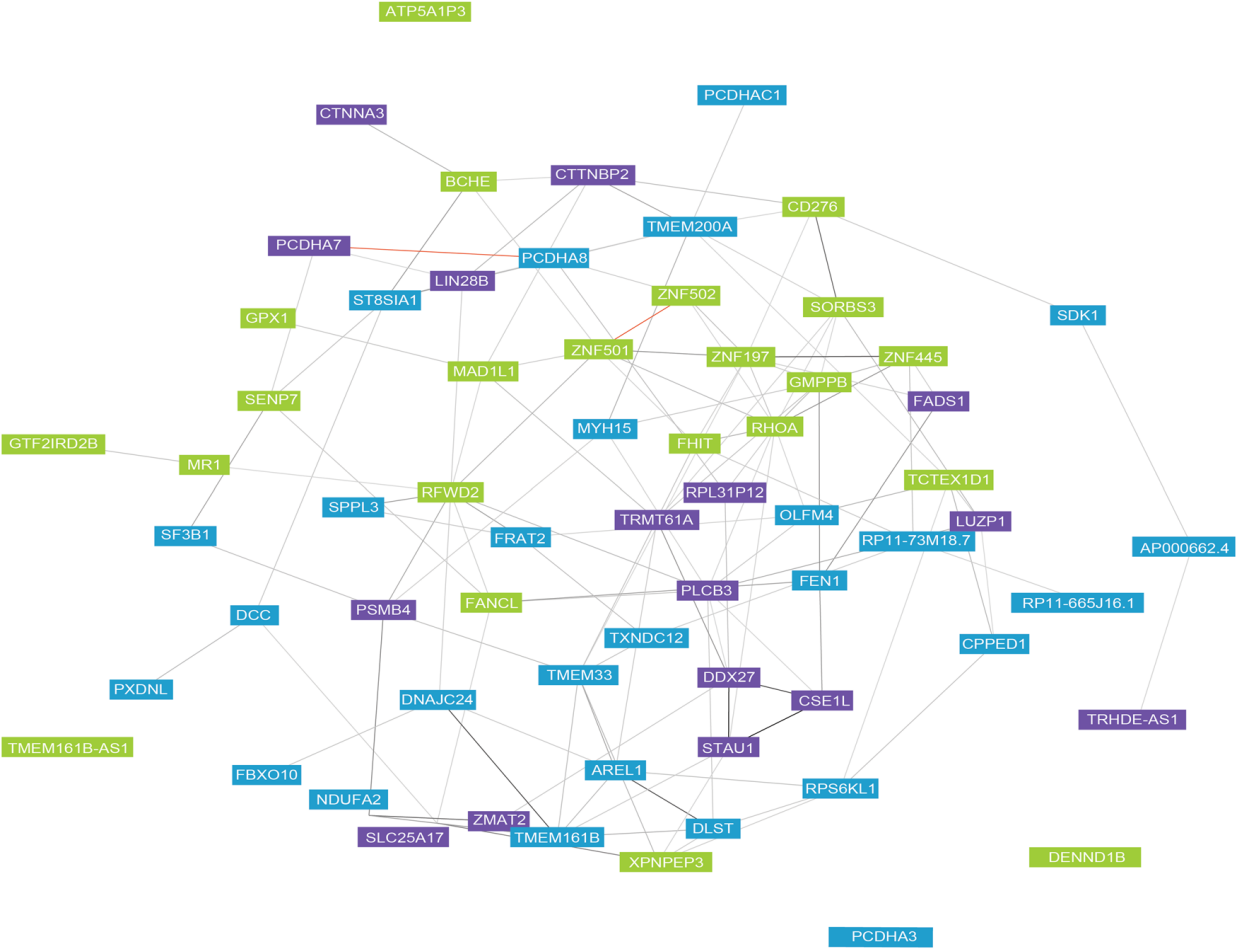
Gene clustering for the identified TWAS genes based on co-expression. Public RNA-sequencing data from 31,499 samples was used to determine co-expression profiles. Co-expression cluster 1 was showed in blue. Co-expression cluster 2 was showed in green. Co-expression cluster 3 was showed in purple. Darker lines represent stronger co-expression.

To detect whether the TWS genes identified by TWAS were enriched in specific pathways, we carried out pathway and gene ontology analysis. All of genes that reached a relaxed Bonferroni-corrected significance were grouped into three different clusters based on co-expression of 31,499 public samples (expression level was quantified with RNA-seq) (Fig. 3). Several biological pathways were significantly enriched among the TWS depression genes, including female pregnancy (Mann–Whitney U-Test, *P* = 8.53 × 10^−6^), formation of Fibrin Clot (Mann–Whitney U-Test, *P* = 7.92 × 10^−5^), cAMP binding (Mann–Whitney U-Test, *P* = 7.45 × 10^−4^) and ephrin signaling (Mann–Whitney U-Test, *P* = 7.37 × 10^−4^) (Table 2). In addition, Human Phenotype Ontology (HPO) analyses suggested that the identified TWAS significant genes were enriched in peripheral axonal degeneration (Mann–Whitney U-Test, *P* = 5.49 × 10^−4^) and neurodevelopmental delay (Mann–Whitney U-Test, *P* = 7.08 × 10^−4^) (Table 2). Interestingly, we noticed that several marginally significant enriched pathways were consistent with previous findings^42^, including G-protein coupled receptor activity (Mann–Whitney U-Test, *P* = 8.54 × 10^−2^).

**Table 2:** Significant pathways of TWAS genes identified through gene-network analysis.

Pathway	Significance	Database
Formation of Fibrin Clot (Clotting Cascade)	7.92E-05	Reactome
Common Pathway of Fibrin Clot Formation	3.98E-04	Reactome
Keratinization	4.95E-04	Reactome
Ephrin signaling	7.37E-04	Reactome
Female pregnancy	8.53E-06	GO Processes
Defense response to Gram-negative bacterium	3.05E-05	GO Processes
Fibrinolysis	3.65E-05	GO Processes
Epithelial to mesenchymal transition	5.63E-04	GO Processes
Pattern specification process	6.96E-04	GO Processes
Protease binding	7.00E-05	GO Function
cAMP binding	7.45E-04	GO Function
Peripheral axonal degeneration	5.49E-04	Human Phenotype Ontology
Neurodevelopmental delay	7.08E-04	Human Phenotype Ontology

### TWS genes showed higher expression level than background genes in the human brain

In addition to pathway enrichment analysis, another important approach to explore the biological function of geneset is the spatio-temporal gene-expression profiling. Based on the expression data from the BrainSpan (http://www.brainspan.org/), we carried out spatio-temporal expression pattern analysis for two TWS genesets (geneset 1: the TWS depression genes identified both in CMC and BrainSeq2 expression panels, *N* = 7; geneset 2: all TWS depression genes across two expression panels, *N* = 53). More detailed information about spatio-temporal expression pattern analysis can be found in the study of Gilman et al.^43^. The average expression level of all TWS depression genes (Supplementary Table 1 and 2) was significantly higher than the expression level of the background genes across all developmental stages (Wilcoxon rank-sum test, *P* < 8.66 × 10^−5^) (Supplementary Fig. 1). Moreover, we found that the expression level of the TWS depression genes was higher in prenatal stage than the postnatal stages in geneset 2 (*P* = 1.30 × 10^−2^, Wilcoxon rank-sum test) but not in geneset 1 (*P* = 6.60 × 10^−2^, Wilcoxon rank-sum test). These data suggest that the identified TWS depression genes may have pivotal roles in brain development and function.

### Cell-type-specific expression analysis of target genes

To explore the expression pattern of the TWS depression genes in different brain cell types, we conducted cell-type-specific expression analyses^39^. TWS depression genes identified in CMC and BrainSeq2 datasets (a total of 53 genes) (Supplementary Table 1 and 2) were analyzed to investigate if TWS depression genes were specifically expressed in specific cell populations. Notably, we found that the TWS depression genes were primarily expressed in two pyramidal cell types (including hippocampal CA1 pyramidal cells and somatosensory pyramidal cells) (i.e. these two pyramidal cell types contained the most numbers of the TWS depression genes that above the specificity score (i.e. specificity score > 0.1)) (Supplementary Fig. 2). This result is consistent with previous findings and provides further support for the involvement of pyramidal cells in depression^44,45^. In addition, this result also suggests that the TWS genes may confer depression risk by affecting the functions of pyramidal cells. To explore the expression pattern of the seven overlapping TWS genes in different human tissues, we performed tissue-type-specific expression analysis using expression data from GTEx. Our results indicated that expression level of *FADS1* and *TCTEX1D1* is low in most human tissues. However, other five genes (*B3GALTL, XPNPEP3, ZMAT2, ZNF501* and *ZNF502*) are all expressed in human brains (Supplementary Figs. 3–9).

### Expression analysis of TWS genes in brains of depression cases and controls

TWAS identified depression-associated genes (Supplementary Tables 1 and 2) under the assumption that genetic variants influence depression risk by modulating gene expression. To further explore if the significant genes identified by TWAS analysis were dysregulated in depression cases compared with controls, we compared the expression level of the TWS genes in brains of depression cases and healthy controls using the expression data from three RNA-seq studies^29–31^. As previous studies have indicated that the DLPFC may play a pivotal role in depression^46,47^, we only selected the expression data from the DLPFC to perform differential expression analysis. These three datasets were GSE102556 (26 depression cases and 22 controls)^29^, GSE101521 (30 depression cases and 29 controls)^30^ and GSE80655 (23 depression cases and 24 controls)^31^. The *P* value was corrected by the Bonferroni correction (for multiple testing) approach, which resulted in a significance threshold of *P* = 9.43 × 10^−4^ (=0.05/53 TWS genes were retained for differential expression analysis analysis). We found that *PCDHA8* (*P* = 1.31 × 10^−3^) was significantly downregulated in depression cases compared with controls in GSE101521 dataset. By contrast, *FANCL* (*P* = 4.88 × 10^−2^) showed a significant upregulation in depression cases compared with controls in GSE101521 dataset (Supplementary Table 3). In GSE80655 dataset, *PCDHA7* showed a significant upregulation (*P* = 4.34 × 10^−3^) (Supplementary Table 3). Interestingly, five TWAS significant genes (*TMEM161B-AS1*, *GMPPB*, *STAU1*, *NDUFA2* and *GPX1*) were significantly upregulated in brains of depression cases compared with controls in GSE102556 dataset (Supplementary Table 3). Taken together, these results further support that the identified TWAS significant genes may have a role in depression. In addition, these results also suggest that the identified risk variants may confer depression risk through regulating gene expression.

## Discussion

Depression is a common psychiatric disorder that is caused by a combination of multiple risk factors, including genetic, psychological, biological and social factors. Although recent GWASs have successfully identified multiple depression risk loci, the biological interpretations and functional understanding of these associations remain poorly understood (partly due to the inability to fine-map depression-relevant genes). TWAS^17^ is a powerful approach to identify associated genes by combining the GWAS results and expression data. In this study, we performed a depression TWAS and identified candidate risk genes for depression. Considering that sample size and tissue matching have pivotal roles in TWAS, we used expression data from the DLPFC (i.e. CMC^20^ and BrainSeq2^21^) to conduct TWAS analysis. We integrated the summary statistics from the largest depression GWAS^14^ so far (*N* = 807,553 individuals) and gene-expression measurements from two large-scale expression studies to identify TWS genes for depression. Overall, we identified 53 significant genes whose expression were associated with depression risk, of which 23 genes did not overlap with a genome-wide significant locus in the depression GWAS.

Our study also highlighted seven overlapping TWS genes (including *B3GALTL*, *FADS1*, *TCTEX1D1*, *XPNPEP3*, *ZMAT2*, *ZNF501* and *ZNF502*) in both CMC and BrainSeq2 datasets. These seven TWS genes may represent high-confidence risk genes for depression as they reached transcriptome-wide significance level in two independent expression datasets. *B3GLCT* encodes Beta 3-Glucosyltransferase, which is involved in metabolism of proteins and O-glycosylation of TSR domain-containing proteins^48^. No previous study showed association between this gene and depression. *TCTEX1D1* (Tctex1 Domain Containing 1) has a role in organelle biogenesis and maintenance, and intraflagellar transport^49^. The protein encoded by *XPNPEP3* belongs to the family of X-pro-aminopeptidases, which remove the N-terminal amino acid from peptides with a proline residue in the penultimate position^50^. *ZMAT2* (Zinc Finger Matrin-Type 2) is related to nucleic acid binding. *ZNF501* (Zinc Finger Protein 501) encodes a DNA-binding transcription factor. An important paralog of *ZNF501* is *ZNF502*. There were no previous studies reported associations between *ZNF501*/*ZNF502* and depression. It is possible that *ZNF501*/*ZNF502* confer risk of depression by regulating gene expression. *FADS1* encodes fatty acid desaturase 1, a protein that is mainly involved in metabolism of alpha-linolenic (omega3), linoleic (omega6) acid and metabolism^51^. Interestingly, a previous study has showed that mRNA expression of *FADS1* was significantly lower in depression patients compared with controls, suggested that *FADS1* may have pivotal roles in depression^52^. More work is needed to characterize the potential role of these genes in depression.

We further performed a depression TWAS analysis using the expression weights data from PsychENCODE^24^. Among the seven overlapping TWS genes, three genes (i.e. *B3GALTL*, *FADS1* and *ZMAT2*) showed significant associations in PsychENCODE dataset (Supplementary Table 4). Spatio-temporal expression pattern analysis showed distinct expression patterns of these seven overlapping TWS genes in the developing and adult human brain (Supplementary Fig. 10). Five genes (*FADS1, XPNPEP3, ZMAT2*, *ZNF501* and *ZNF502*) showed higher expression in the prenatal developing human brain than adult brain. However, other two genes (*B3GALTL* and *TCTEX1D1*) showed reverse spatio-temporal expression patterns.

Of note, we noticed that previous studies^11,53^ also used CMC dataset as SNP-expression weights for depression TWAS. To identify genes whose genetically regulated expression are associated with complex diseases and traits, Mancuso et al. performed a comprehensive TWAS by integrating multiple expression references and GWAS summary statistics^53^. They identified over 1000 genes whose expression are associated with diseases or traits. Based on this comprehensive TWAS, they developed TWAS hub (http://twas-hub.org/), which includes multiple TWAS results and provides a convenient online resource to explore if a specific gene is associated with disease or trait. However, the sample size (i.e. depression cases and controls) included in this study is relatively small (*N* = 135,458 depression cases and 344,901 controls). We used the summary statistics from a larger depression GWAS (*N* = 246,363 depression cases and 561,190 controls) in this study. In addition, we also utilized another independent expression weights dataset (BrainSeq2) in this study.

We also explored the ethnic difference for the TWAS or GWAS results for major depressive disorder across populations (i.e. Caucasian v.s. Asian). We compared the depression GWAS performed in Asian (CONVERGE^9^) and European populations^14^. The CONVERGE consortium showed that rs12415800 (*P* = 1.92 × 10^−8^) and rs35936514 (*P* = 1.27 × 10^−8^) were significantly associated with depression in Chinese population^9^. However, recent large-scale GWAS did not found significant associations between these risk variants and depression in European populations^14^. The frequencies of the risk alleles of the identified risk variants (rs12415800 and rs35936514) show dramatic differences in Chinese and European populations, suggesting the potential ethnic difference (i.e. population heterogeneity) of depression risk variants. We further performed a TWAS using expression weights data^25^ and GWAS^9^ of the Asians. We did not identify any TWS genes after Bonferroni correction (*P* < 7.32 × 10^−5^) (Supplementary Table 5). However, it should be noted that the sample size included in eQTL and GWAS were relatively small. In addition, as there is no public available brain eQTL of Asians, we used eQTL data from lymphoblastoid cell lines. More work is needed to explore the ethnic difference for the TWAS or GWAS results for major depressive disorder across populations (i.e. Caucasian v.s. Asian).

To explore if the three TWS genes (i.e. *ZNF501*, *ZNF502* and *B3GALTL*) for depression were also associated with schizophrenia and bipolar disorder, we first compared the genetic results of these three TWS genes in GWAS of depression^14^, bipolar disorder^54^ and schizophrenia^55^. These TWS genes did not show significant associations with bipolar disorder and schizophrenia (Supplementary Fig. 11). To explore if expression of *ZNF501*, *ZNF502*, *B3GALTL* were associated with schizophrenia and bipolar disorder, we further examined TWAS results of these three genes (i.e. *ZNF501*, *ZNF502* and *B3GALTL*) in PsychENCODE^24^ (Supplementary Table 6). *ZNF501*, *ZNF502* and *B3GALTL* did not show TWS associations with schizophrenia and bipolar disorder, suggesting that the association is depression specific. More work is needed to investigate the role of these genes in depression.

In addition to the seven overlapping TWS genes, other genes may also have a role in depression. For example, although eight genes (*PCDHA8, FANCL, TMEM161B-AS1, GMPPB, STAU1, NDUFA2, GPX1* and *PCDHA7*) were not included in the overlapping TWS gene list, we noticed that these genes were significantly dysregulated in the DLPFC of depression cases compared with controls (Supplementary Table 3), suggesting the potential role of these genes in depression. Further work is needed to investigate the potential role of these genes in depression.

The majority of TWS depression genes identified in our study are located around known GWAS loci. Interestingly, conditional and joint analyses (conditioning on the top TWAS gene) demonstrated that several of the genome-wide significant signals from the depression GWAS were driven by the TWAS expression signals. There was little residual association signal from the genetic variant in the GWAS locus after conditioning on the predicted expression signals. Notably, our TWAS analysis suggested that the *FADS1* gene may represent a promising candidate for depression (as the length of *FADS1* was small compared with nearby genes, these smaller genes are often overlooked in GWAS because there are many larger protein-coding genes nearby). Our TWAS results showed that the expression of *FADS1* largely explains the GWAS signal of depression, suggesting the power of TWAS to detect promising target genes. Previous study has showed that the mRNA expression of *FADS1* was significantly downregulated in the postmortem prefrontal cortex of major depression patients compared with controls^52^. Another microarray study also found that *FADS1* expression was downregulated in the prefrontal cortex of suicidal male major depression patients^56^. These results suggest that *FADS1* implicated in TWAS may represent a novel risk gene for depression.

In addition to identifying the candidate genes for depression, pathway enrichment analysis was also performed to better understand the biological implications of these TWAS significant genes in the context of the biological processes. Our pathways analysis identified several interesting pathways for depression, including female pregnancy, formation of Fibrin Clot, cAMP binding and ephrin signaling. Besides, our pathway analysis also confirmed previously identified pathways for depression. Among these enrichment pathways, synaptic transmission (Mann–Whitney U-Test, *P* = 1.94 × 10^−2^), dopaminergic (Mann–Whitney U-Test, *P* = 1.94 × 10^−2^) and G-protein coupled receptor signaling pathway (Mann–Whitney U-Test, *P* = 5.01 × 10^−2^) have been reported to be implicated in the pathogenesis of depression^57–59^. Finally, we further performed the differential expression analysis for the identified TWS genes across three depression datasets (GSE102556^29^, GSE101521^30^ and GSE80655^31^). Several TWAS significant genes were also dysregulated in brains of depression cases compared with controls (including *PCDHA8*, *FANCL*, *TMEM161B-AS1*, *GMPPB*, *STAU1*, *NDUFA2*, *GPX1* and *PCDHA7*), implying that genetic variants may contribute to depression risk by regulating gene expression.

There were several limitations in this study. First, TWAS typically restricts to impute the cis genetic component of expression on traits, thus, variants influencing depression but are independent of cis expression will not be identified. Second, the number of identified TWAS genes is limited by the size of the training cohort (i.e. reference individuals with expression and genotype measured data) and the quality of the training data. Therefore, more work is needed to increase the sample size and quality of expression data for future TWAS analysis. Finally, the summary-based TWAS could not pinpoint the causal variants and genes for depression. More works are needed to pinpoint the causal variants and to elucidate their roles in depression pathogenesis.

In summary, we performed a depression TWAS based on the integration of depression GWAS summary statistics and gene-expression data from the DLPFC. We identified promising candidate susceptibility genes for depression. Further functional characterization of the identified TWS genes will provide pivotal information for understanding the etiology of depression, facilitating biological interpretations of depression GWAS results and prioritizing potential targets for drug development.

## Supplementary information

Supplementary Material

## Data Availability

All data relevant to the study are included in the article or uploaded as online supplementary information. The data generated in this study will be available from the corresponding author on reasonable request.
