# Supplementary material for: Transcriptome-wide association study identifies new susceptibility genes and pathways for depression
Source: Transl Psychiatry. 2021 May 21;11:306. doi: 10.1038/s41398-021-01411-w (PMC8140098; doi:10.1038/s41398-021-01411-w)
Supplement: Supplementary file 1 — Supplementary Material [file 41398_2021_1411_MOESM1_ESM.docx]

**Supplementary Material for**

**Transcriptome-wide association study identifies new susceptibility genes and pathways for depression**

Xiaoyan Li^1,2^, Xi Su^3,4^, Jiewei Liu^1^, Huijuan Li^1^, Ming Li^1,5^, the 23andMe Research Team^6^, Wenqiang Li^3,4^ & Xiong-Jian Luo^1,5,7^

**Supplementary Table 1. Transcriptome-wide significant depression risk genes identified in CMC data set**

| **Implicated genes** | **Tissue** | **Chr** | **Best eQTL^a^** | **Direction**  **Z-score^b^** | **TWAS**  **P-value** | **Implicated in**  **2019 depression GWAS** |
| --- | --- | --- | --- | --- | --- | --- |
| *DENND1B* | DLPFC | 1 | rs16841842 | 6.77 | 1.33E-11 | Yes |
| *DLST* | DLPFC | 14 | rs2111705 | 6.47 | 9.61E-11 | Yes |
| *ZNF445* | DLPFC | 3 | rs7616113 | -6.42 | 1.41E-10 | Yes |
| *LIN28B* | DLPFC | 6 | rs4946651 | -6.35 | 2.18E-10 | Yes |
| *TRMT61A* | DLPFC | 14 | rs942866 | 6.34 | 2.24E-10 | Yes |
| *CD276* | DLPFC | 15 | rs7176654 | -6.05 | 1.42E-09 | No |
| *XPNPEP3* | DLPFC | 22 | rs138354 | 5.93 | 2.97E-09 | Yes |
| *FANCL* | DLPFC | 2 | rs11682175 | -5.83 | 5.39E-09 | Yes |
| *TCTEX1D1* | DLPFC | 1 | rs512691 | -5.81 | 6.26E-09 | Yes |
| *NDUFA2* | DLPFC | 5 | rs12659980 | 5.58 | 2.36E-08 | No |
| *TXNDC12* | DLPFC | 1 | rs1538881 | -5.56 | 2.76E-08 | No |
| *FBXO10* | DLPFC | 9 | rs12237351 | -5.36 | 8.36E-08 | No |
| *ZNF197* | DLPFC | 3 | rs2372688 | -5.27 | 1.38E-07 | Yes |
| *ZMAT2* | DLPFC | 5 | rs801183 | -5.24 | 1.65E-07 | No |
| *DDX27* | DLPFC | 20 | rs7266044 | 5.15 | 2.64E-07 | No |
| *SF3B1* | DLPFC | 2 | rs3792159 | 5.13 | 2.90E-07 | No |
| *OLFM4* | DLPFC | 13 | rs1535576 | 5.04 | 4.71E-07 | Yes |
| *PCDHA7* | DLPFC | 5 | rs12659980 | -5.02 | 5.04E-07 | No |
| *PCDHAC1* | DLPFC | 5 | rs753279 | -5.02 | 5.07E-07 | No |
| *SLC25A17* | DLPFC | 22 | rs80533 | -4.97 | 6.74E-07 | Yes |
| *ZNF502* | DLPFC | 3 | rs10514710 | -4.93 | 8.21E-07 | Yes |
| *GPX1* | DLPFC | 3 | rs4955430 | 4.87 | 1.14E-06 | Yes |
| *RPS6KL1* | DLPFC | 14 | rs2286913 | -4.85 | 1.24E-06 | Yes |
| *CSE1L* | DLPFC | 20 | rs2075679 | -4.85 | 1.25E-06 | No |
| *FADS1* | DLPFC | 11 | rs174568 | -4.82 | 1.42E-06 | No |
| *FEN1* | DLPFC | 11 | rs174568 | 4.80 | 1.62E-06 | No |
| *B3GALTL* | DLPFC | 13 | rs4065552 | -4.79 | 1.70E-06 | Yes |
| *PSMB4* | DLPFC | 1 | rs6587572 | -4.74 | 2.13E-06 | No |
| *ZNF501* | DLPFC | 3 | rs10514710 | -4.69 | 2.68E-06 | Yes |
| *DNAJC24* | DLPFC | 11 | rs17632324 | 4.68 | 2.87E-06 | Yes |
| *FRAT2* | DLPFC | 10 | rs7101333 | -4.68 | 2.92E-06 | No |
| *RHOA* | DLPFC | 3 | rs4955430 | 4.67 | 2.99E-06 | Yes |
| *GMPPB* | DLPFC | 3 | rs6809879 | 4.63 | 3.70E-06 | Yes |

^a^The SNP with the best eQTL in the locus.

^b^The TWAS effect-size (i.e., Z-score) is an estimate of the genetic covariance between gene expression and the GWAS trait, and the direction of effect is informative of this relationship. A positive Z-score suggests that over-expression of the gene leads to an increase in the phenotype (and vice versa).

**Supplementary Table 2. Transcriptome-wide significant depression risk genes identified in BrainSeq2 data set**

| **Implicated genes** | **Tissue** | **Chr** | **Best eQTL^a^** | **Direction Z-score^b^** | **TWAS**  **P-value** | **Implicated in**  **2019 depression GWAS** |
| --- | --- | --- | --- | --- | --- | --- |
| *RPL31P12* | DLPFC | 1 | rs3101336 | -10.04 | 1.04E-23 | Yes |
| *TMEM161B-AS1* | DLPFC | 5 | rs16903057 | 6.85 | 7.31E-12 | Yes |
| *TMEM161B* | DLPFC | 5 | rs6882046 | -6.65 | 2.87E-11 | Yes |
| *RFWD2* | DLPFC | 1 | rs2039230 | -6.55 | 5.82E-11 | Yes |
| *AREL1* | DLPFC | 14 | rs752857 | -6.07 | 1.28E-09 | Yes |
| *CTTNBP2* | DLPFC | 7 | rs989996 | -6.04 | 1.51E-09 | Yes |
| *RP11-73M18.7* | DLPFC | 14 | rs3742465 | 6.02 | 1.76E-09 | Yes |
| *B3GALTL* | DLPFC | 13 | rs9543390 | -5.52 | 3.44E-08 | Yes |
| *GTF2IRD2B* | DLPFC | 7 | rs1635759 | -5.51 | 3.53E-08 | No |
| *TCTEX1D1* | DLPFC | 1 | rs10493416 | -5.49 | 3.99E-08 | Yes |
| *DCC* | DLPFC | 18 | rs11665242 | 5.47 | 4.55E-08 | Yes |
| *PCDHA8* | DLPFC | 5 | rs10038174 | -5.22 | 1.79E-07 | No |
| *XPNPEP3* | DLPFC | 22 | rs2899341 | 5.18 | 2.26E-07 | Yes |
| *FADS1* | DLPFC | 11 | rs174566 | -5.12 | 3.08E-07 | No |
| *FHIT* | DLPFC | 3 | rs12053817 | -5.08 | 3.86E-07 | Yes |
| *PCDHA3* | DLPFC | 5 | rs2531349 | -5.07 | 4.03E-07 | No |
| *ZMAT2* | DLPFC | 5 | rs3756341 | -5.02 | 5.28E-07 | No |
| *SDK1* | DLPFC | 7 | rs17133289 | -4.98 | 6.47E-07 | No |
| *ZNF502* | DLPFC | 3 | rs10514710 | -4.98 | 6.50E-07 | Yes |
| *ATP5A1P3* | DLPFC | 16 | rs1424241 | -4.95 | 7.33E-07 | No |
| *CPPED1* | DLPFC | 16 | rs8048866 | 4.90 | 9.81E-07 | No |
| *RP11-665J16.1* | DLPFC | 15 | rs4886574 | -4.80 | 1.59E-06 | No |
| *ZNF501* | DLPFC | 3 | rs10514710 | -4.79 | 1.68E-06 | Yes |
| *TMEM33* | DLPFC | 4 | rs13122139 | 4.76 | 1.91E-06 | Yes |
| *BCHE* | DLPFC | 3 | rs9873779 | 4.70 | 2.57E-06 | No |
| *MAD1L1* | DLPFC | 7 | rs17186327 | 4.70 | 2.58E-06 | Yes |
| *STAU1* | DLPFC | 20 | rs2295714 | -4.64 | 3.55E-06 | No |

^a^The SNP with the best eQTL in the locus.

^b^The TWAS effect-size (i.e., Z-score) is an estimate of the genetic covariance between gene expression and the GWAS trait, and the direction of effect is informative of this relationship. A positive Z-score suggests that over-expression of the gene leads to an increase in the phenotype (and vice versa).

**Supplementary Table 3. Differential expression analysis based on three RNA-seq datasets**

| **Gene** | **Location** | **Significance** | ***P* adjust^a^** | **Database** |
| --- | --- | --- | --- | --- |
| *PCDHA8* | chr5:140220907-140391929 | 1.31E-03 | NS | GSE101521 |
| *FANCL* | chr2:58386378-58468507 | 4.88E-02 | NS | GSE101521 |
| *TMEM161B-AS1* | chr5:87564712-87732502 | 1.08E-02 | NS | GSE102556 |
| *GMPPB* | chr3:49754277-49761384 | 2.09E-02 | NS | GSE102556 |
| *STAU1* | chr20:47729878-47804904 | 2.35E-02 | NS | GSE102556 |
| *NDUFA2* | chr5:140018325-140027370 | 3.33E-02 | NS | GSE102556 |
| *GPX1* | chr3:49394609-49396033 | 4.95E-02 | NS | GSE102556 |
| *PCDHA7* | chr5:140213969-140391929 | 4.34E-03 | NS | GSE80655 |

^a^*P* value was adjusted for multiple testing (i.e., Bonferroni corrected threshold of *P* = 9.43 × 10^−4^ was used). NS, not significant.

**Supplementary Table 4. TWS genes with a *P* < 3.39 × 10^-6^ for depressions in PsychENCODE dataset**

| **Gene** | | **Chr** | **Start** | **End** | **Best eQTL** | **TWAS.Z** | **TWAS.P** |
| --- | --- | --- | --- | --- | --- | --- | --- |
| *RPL31P12* | 1 | 72767155 | 72767512 | 1:72812249 | -10.44 | 1.59E-25 |  |
| *ZSCAN12P1* | 6 | 28058932 | 28061442 | 6:28296842 | 9.54 | 1.40E-21 |  |
| *HIST1H4L* | 6 | 27840926 | 27841289 | 6:27771106 | 8.77 | 1.86E-18 |  |
| *TMEM106B* | 7 | 12250867 | 12282993 | 7:12262242 | -7.67 | 1.69E-14 |  |
| *AREL1* | 14 | 75120140 | 75179818 | 14:75120628 | -7.53 | 5.10E-14 |  |
| *RP5-874C20.3* | 6 | 28234788 | 28245974 | 6:28070998 | 7.52 | 5.29E-14 |  |
| *PIGFP2* | 9 | 126605315 | 126605965 | 9:126658613 | -7.49 | 6.65E-14 |  |
| *RP11-73M18.6* | 14 | 104153913 | 104154464 | 14:104144941 | 7.22 | 5.26E-13 |  |
| *RP1-265C24.5* | 6 | 28083406 | 28084329 | 6:28054404 | 7.00 | 2.64E-12 |  |
| *RP11-600F24.2* | 14 | 103878456 | 103879098 | 14:104009939 | 6.98 | 3.05E-12 |  |
| *RAB27B* | 18 | 52385091 | 52562747 | 18:52472982 | 6.94 | 3.81E-12 |  |
| *RP11-73M18.7* | 14 | 104160897 | 104161507 | 14:104091115 | 6.93 | 4.34E-12 |  |
| *TMEM161B-AS1* | 5 | 87564712 | 87732502 | 5:87608635 | 6.71 | 1.91E-11 |  |
| *NKAPL* | 6 | 28227098 | 28228736 | 6:28217931 | 6.64 | 3.04E-11 |  |
| *TCF4* | 18 | 52889562 | 53332018 | 18:52881614 | -6.57 | 5.00E-11 |  |
| *HIST1H4J* | 6 | 27791884 | 27792257 | 6:27708732 | 6.49 | 8.36E-11 |  |
| *CTC-467M3.3* | 5 | 87988462 | 87989789 | 5:87986284 | -6.47 | 9.58E-11 |  |
| *RP11-73M18.9* | 14 | 104179904 | 104180586 | 14:104091434 | 6.46 | 1.08E-10 |  |
| *RP1-313I6.12* | 6 | 28046570 | 28048908 | 6:28065993 | 6.45 | 1.09E-10 |  |
| *DLST* | 14 | 75348594 | 75370448 | 14:75355203 | 6.34 | 2.29E-10 |  |
| *PRSS16* | 6 | 27215480 | 27224403 | 6:27121721 | -6.32 | 2.55E-10 |  |
| *B3GALTL* | 13 | 31774073 | 31906413 | 13:31854693 | -6.31 | 2.87E-10 |  |
| *RP11-318C24.2* | 1 | 175873898 | 175889649 | 1:176083411 | -6.29 | 3.16E-10 |  |
| *RP6-109B7.3* | 22 | 46449585 | 46453090 | 22:46421145 | 6.22 | 5.01E-10 |  |
| *TRMT61A* | 14 | 103995521 | 104003410 | 14:104001517 | 6.07 | 1.29E-09 |  |
| *LIN28B* | 6 | 105404923 | 105531207 | 6:105382137 | -6.05 | 1.43E-09 |  |
| *HMGN4* | 6 | 26538633 | 26546482 | 6:26566965 | -6.04 | 1.57E-09 |  |
| *RPS6KL1* | 14 | 75370657 | 75390099 | 14:75292662 | -5.98 | 2.17E-09 |  |
| *DENND1A* | 9 | 126141933 | 126692431 | 9:126703738 | 5.95 | 2.65E-09 |  |
| *AC068490.1* | 2 | 22167481 | 22188423 | 2:22491522 | 5.74 | 9.30E-09 |  |
| *CHD6* | 20 | 40030741 | 40247133 | 20:40181092 | -5.69 | 1.31E-08 |  |
| *ZNF192P1* | 6 | 28129559 | 28137372 | 6:28112713 | -5.67 | 1.41E-08 |  |
| *ZC3H7B* | 22 | 41697526 | 41756151 | 22:41753603 | 5.63 | 1.78E-08 |  |
| *CTC-329D1.3* | 5 | 139728069 | 139737379 | 5:140131786 | 5.63 | 1.84E-08 |  |
| *RANGAP1* | 22 | 41641615 | 41682255 | 22:41665722 | -5.56 | 2.67E-08 |  |
| *HNRNPA1P46* | 1 | 191115155 | 191116091 | 1:191510852 | -5.49 | 4.03E-08 |  |
| *ZKSCAN8* | 6 | 28109688 | 28127250 | 6:28112713 | -5.46 | 4.83E-08 |  |
| *PCDHA7* | 5 | 140213969 | 140391929 | 5:140203432 | -5.45 | 4.97E-08 |  |
| *PCDHA8* | 5 | 140220907 | 140391929 | 5:140145206 | -5.36 | 8.31E-08 |  |
| *TSPAN31* | 12 | 58131796 | 58143994 | 12:58142854 | 5.30 | 1.15E-07 |  |
| *RP11-73M18.8* | 14 | 104162690 | 104163500 | 14:104144941 | 5.24 | 1.64E-07 |  |
| *SLC30A9* | 4 | 41992489 | 42092474 | 4:42048091 | -5.23 | 1.73E-07 |  |
| *PCDHA2* | 5 | 140174444 | 140391929 | 5:140136804 | -5.21 | 1.93E-07 |  |
| *ZNF638* | 2 | 71503691 | 71662199 | 2:71521191 | -5.15 | 2.62E-07 |  |
| *ZBTB45* | 19 | 59024897 | 59050278 | 19:59013196 | 5.13 | 2.85E-07 |  |
| *LINC00899* | 22 | 46435787 | 46440733 | 22:46438611 | -5.09 | 3.52E-07 |  |
| *DCC* | 18 | 49866542 | 51057784 | 18:50697120 | 5.08 | 3.76E-07 |  |
| *GPX1* | 3 | 49394609 | 49396033 | 3:49368317 | 5.08 | 3.77E-07 |  |
| *PSMB4* | 1 | 151372010 | 151374420 | 1:151374545 | -5.07 | 3.99E-07 |  |
| *RERE* | 1 | 8412457 | 8877702 | 1:8468278 | -5.04 | 4.59E-07 |  |
| *RSRC1* | 3 | 157823644 | 158263519 | 3:158263154 | 4.99 | 6.01E-07 |  |
| *SMIM2-AS1* | 13 | 44716682 | 44813010 | 13:44711625 | 4.98 | 6.42E-07 |  |
| *CTD-2298J14.2* | 14 | 42057064 | 42074059 | 14:42433768 | 4.96 | 7.01E-07 |  |
| *CSNK1G1* | 15 | 64457716 | 64648442 | 15:64661937 | -4.95 | 7.53E-07 |  |
| *PRSS51* | 8 | 10340388 | 10405095 | 8:10252577 | -4.91 | 9.14E-07 |  |
| *SF3B1* | 2 | 198254508 | 198299815 | 2:198277551 | 4.91 | 9.16E-07 |  |
| *NMU* | 4 | 56461396 | 56502865 | 4:56289778 | 4.89 | 1.03E-06 |  |
| *PMFBP1* | 16 | 72146056 | 72210777 | 16:72248130 | -4.87 | 1.11E-06 |  |
| *NDUFA2* | 5 | 140018325 | 140027370 | 5:140032947 | 4.86 | 1.15E-06 |  |
| *SENP7* | 3 | 101043049 | 101232085 | 3:101140859 | 4.86 | 1.16E-06 |  |
| *AL022393.7* | 6 | 28143966 | 28144452 | 6:28326490 | -4.83 | 1.36E-06 |  |
| *RP1-153G14.4* | 6 | 27371789 | 27374743 | 6:27426717 | 4.82 | 1.46E-06 |  |
| *AC016629.3* | 19 | 59105263 | 59110722 | 19:59028585 | 4.82 | 1.47E-06 |  |
| *TMEM258* | 11 | 61535973 | 61560274 | 11:61551927 | 4.81 | 1.52E-06 |  |
| *FADS1* | 11 | 61567099 | 61596790 | 11:61575158 | -4.79 | 1.64E-06 |  |
| *RP13-996F3.3* | 15 | 83141718 | 83182928 | 15:83097970 | -4.79 | 1.67E-06 |  |
| *NRXN2* | 11 | 64373646 | 64490660 | 11:64471986 | -4.77 | 1.81E-06 |  |
| *ZSCAN16* | 6 | 28092338 | 28097860 | 6:28092603 | 4.77 | 1.82E-06 |  |
| *BAG5* | 14 | 104022881 | 104029168 | 14:104046834 | -4.77 | 1.84E-06 |  |
| *RP11-159N11.4* | 11 | 113239200 | 113239839 | 11:113187949 | -4.74 | 2.18E-06 |  |
| *CDH9* | 5 | 26880709 | 27121257 | 5:26983657 | -4.72 | 2.31E-06 |  |
| *RHOA* | 3 | 49396578 | 49450431 | 3:49393409 | 4.71 | 2.44E-06 |  |
| *TRIM8* | 10 | 104404253 | 104418164 | 10:104420754 | 4.68 | 2.90E-06 |  |
| *RP11-397A16.2* | 18 | 53443958 | 53448952 | 18:53455685 | -4.67 | 3.06E-06 |  |

*Bonferroni-corrected *P* threshold (i.e., *P* = 3.39 × 10^-6^ (0.05/14,742))

**Supplementary Table 5. Top TWS depression genes identified using the GWAS and eQTL data of Asians**

| **Gene** | **Region** | **Best eQTL** | **TWAS.Z** | **TWAS.P** |
| --- | --- | --- | --- | --- |
| *FYN* | chr6:111981535-112194655 | rs17073074 | 3.21 | 1.34E-03 |
| *TCP11* | chr6:35085848-35116387 | rs820099 | 3.13 | 1.75E-03 |
| *RETSAT* | chr2:85569211-85581743 | rs1877954 | -3.00 | 2.73E-03 |
| *C1orf74* | chr1:209955661-209957904 | rs11119822 | -2.98 | 2.91E-03 |
| *BICD1* | chr12:32259769-32536567 | rs11831258 | 2.88 | 3.94E-03 |
| *OR7D2* | chr19:9296279-9299493 | rs13345998 | -2.83 | 4.72E-03 |
| *BTN3A2* | chr6:26365387-26378546 | rs9393713 | -2.77 | 5.63E-03 |

**Supplementary Table 6. Schizophrenia and bipolar disorder TWAS results for the seven overlapping TWS genes (in PsychENCODE dataset)**

| **Gene** | **Region** | **Schizophrenia (Clozuk.2018)** | |  | **Bipolar disorder (PGC.2018)** | |
| --- | --- | --- | --- | --- | --- | --- |
|  |  | **TWAS.Z** | **TWAS.P** |  | **TWAS.Z** | **TWAS.P** |
| *ZNF501* | chr3:44771088-44778575 | -0.52 | 6.02E-01 |  | -2.94 | 3.25E-03 |
| *ZNF502* | chr3:44754135-44765323 | -0.29 | 7.73E-01 |  | NA | NA |
| *B3GALTL* | chr13:31774073-31906413 | 1.68 | 9.26E-02 |  | 2.22 | 2.62E-02 |


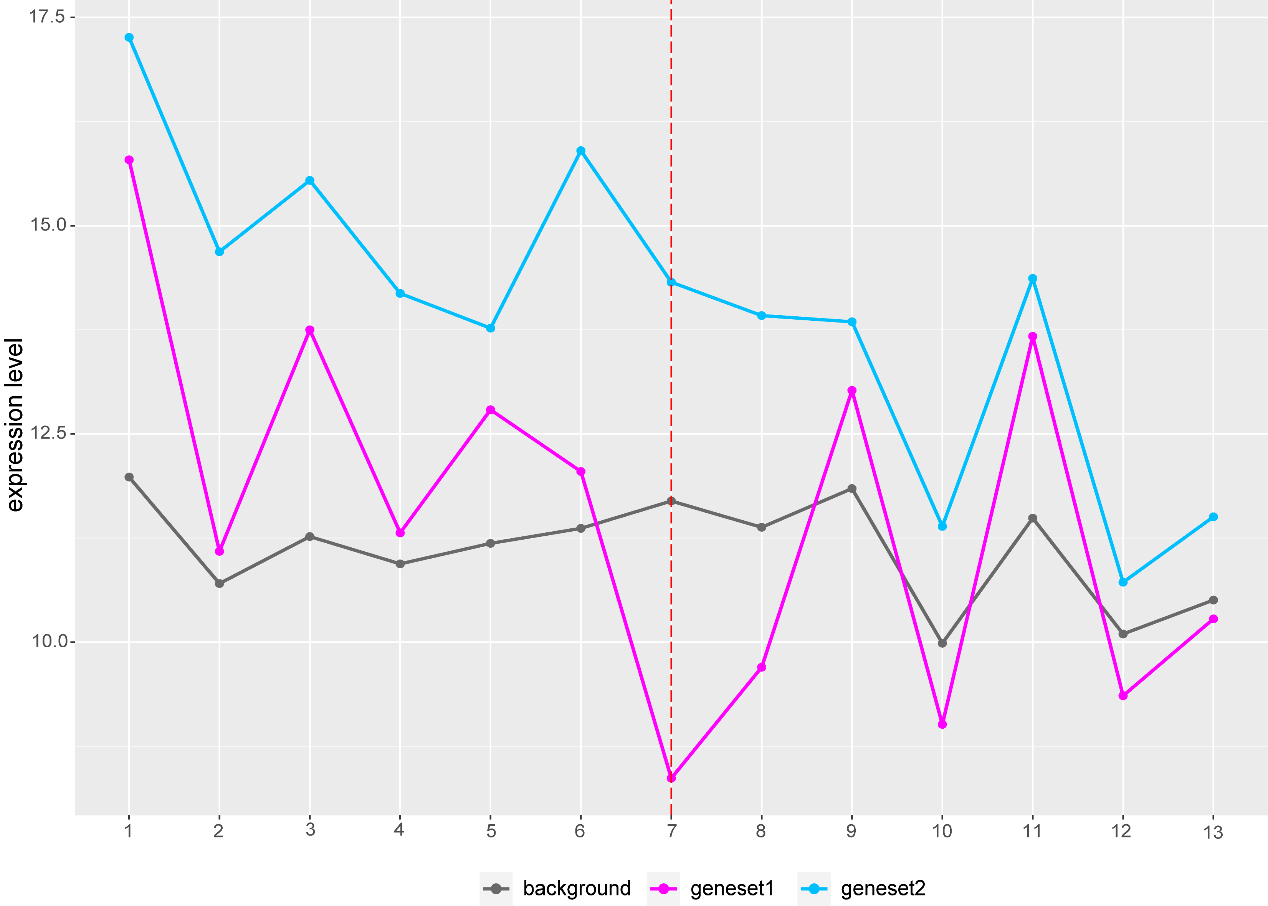


**Supplementary Figure 1. Expression of the transcriptome-wide significant depression risk genes across different developmental stages of the human brain.** Data were downloaded from the BrainSpan (<http://www.brainspan.org/>)[^1^](#_ENREF_1). Development stages were as follows: stage 1: Embryonic (4–8 PCW, postconception week); stage 2: Early fetal1(8–10 PCW); stage 3: Early fetal2 (10–13 PCW); stage 4: Early mid-fetal1 (13–16 PCW); stage 5: Early mid-fetal2 (16–19 PCW); stage 6: Late mid-fetal (19–24 PCW); stage 7: Late fetal (24–38 PCW); stage 8: Neonatal & early infancy (0–6 M, Month); stage 9: Late infancy (6–12 M); stage 10: Early childhood (1–6 Y, Year); stage 11: Middle and late childhood (6–12 Y); stage 12: Adolescence (12–20 Y); stage 13: Young adulthood (20–40 Y).


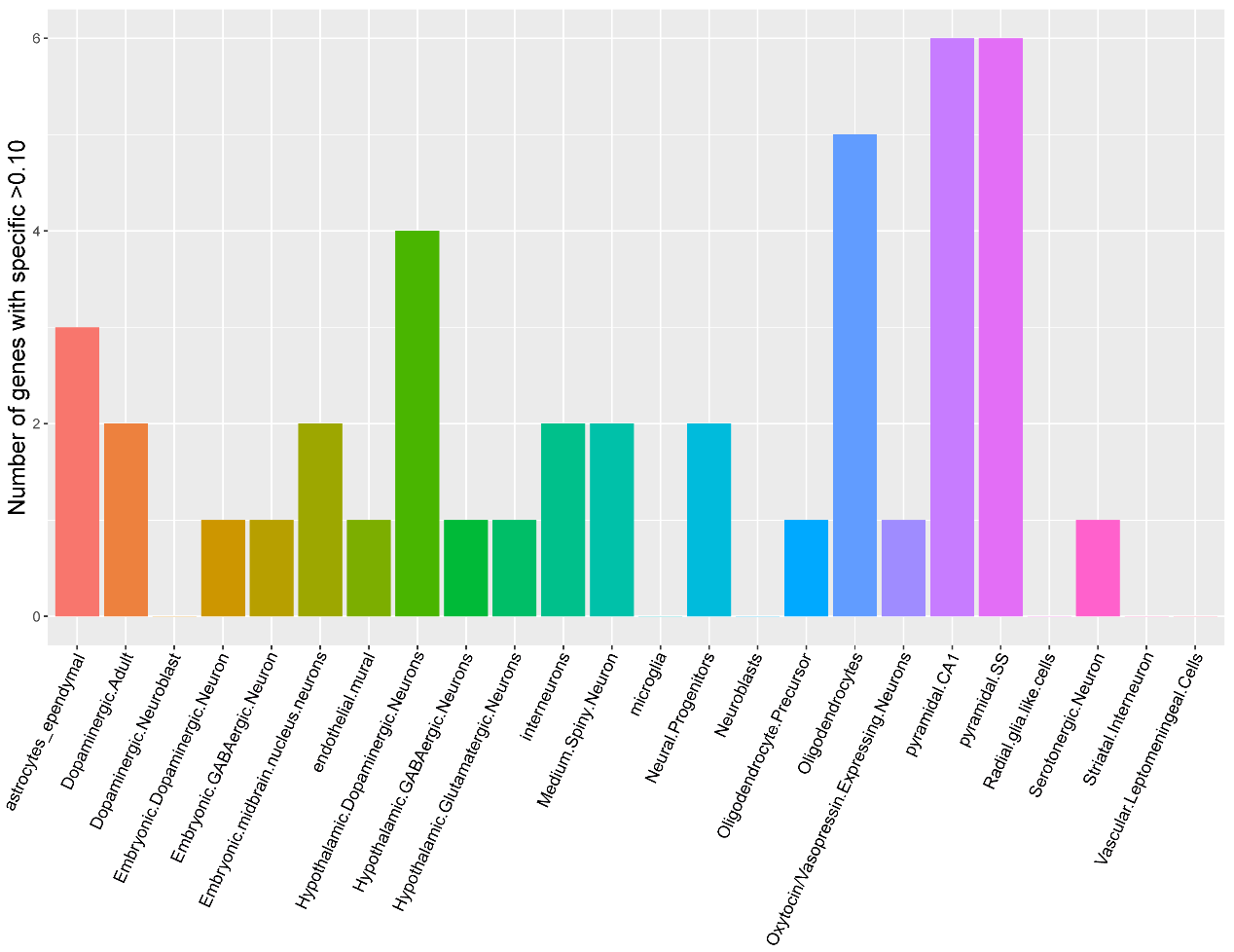


**Supplementary Figure 2. Transcriptome-wide significant depression risk genes were mainly expressed in pyramidal cells.** The cell-type specific expression pattern data were obtained from Skene et al.[^2^](#_ENREF_2)


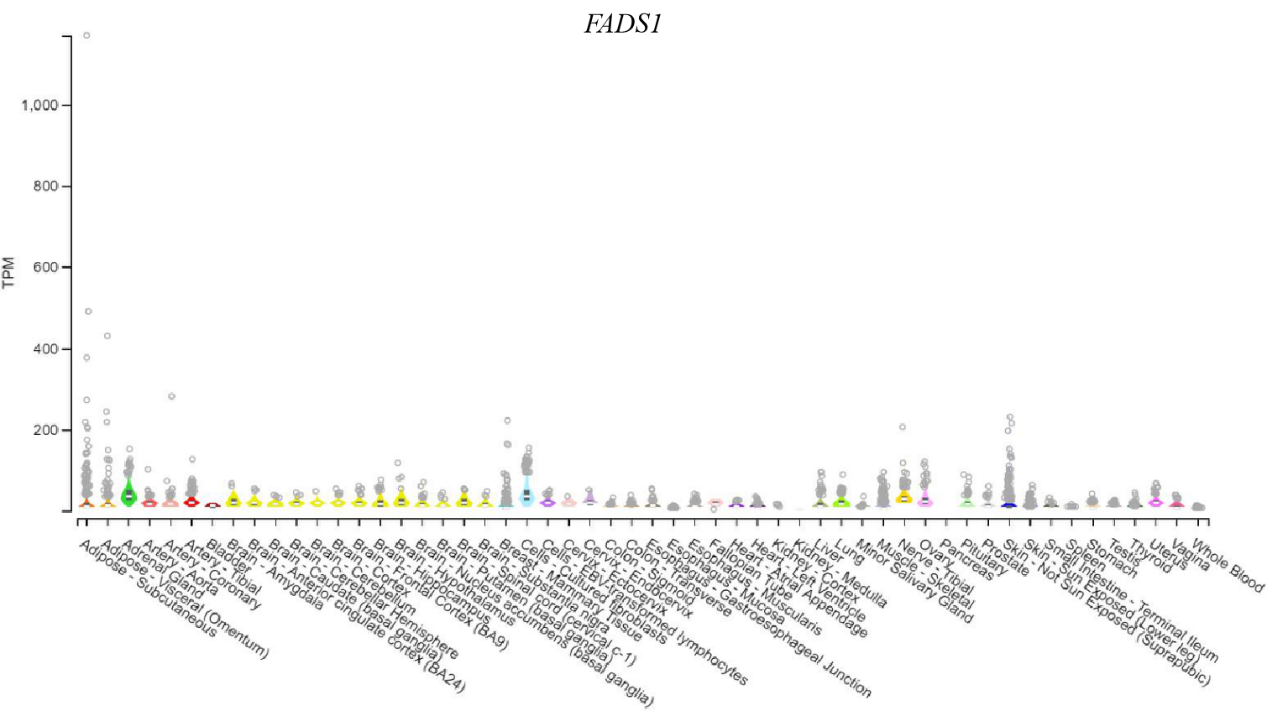


**Supplementary Figure 3. Expression of TWS gene *FADS1* in human tissues (data from the GTEx^[3](#_ENREF_3)^).** The x axis shows 54 tissue types. One color indicates one tissue group.


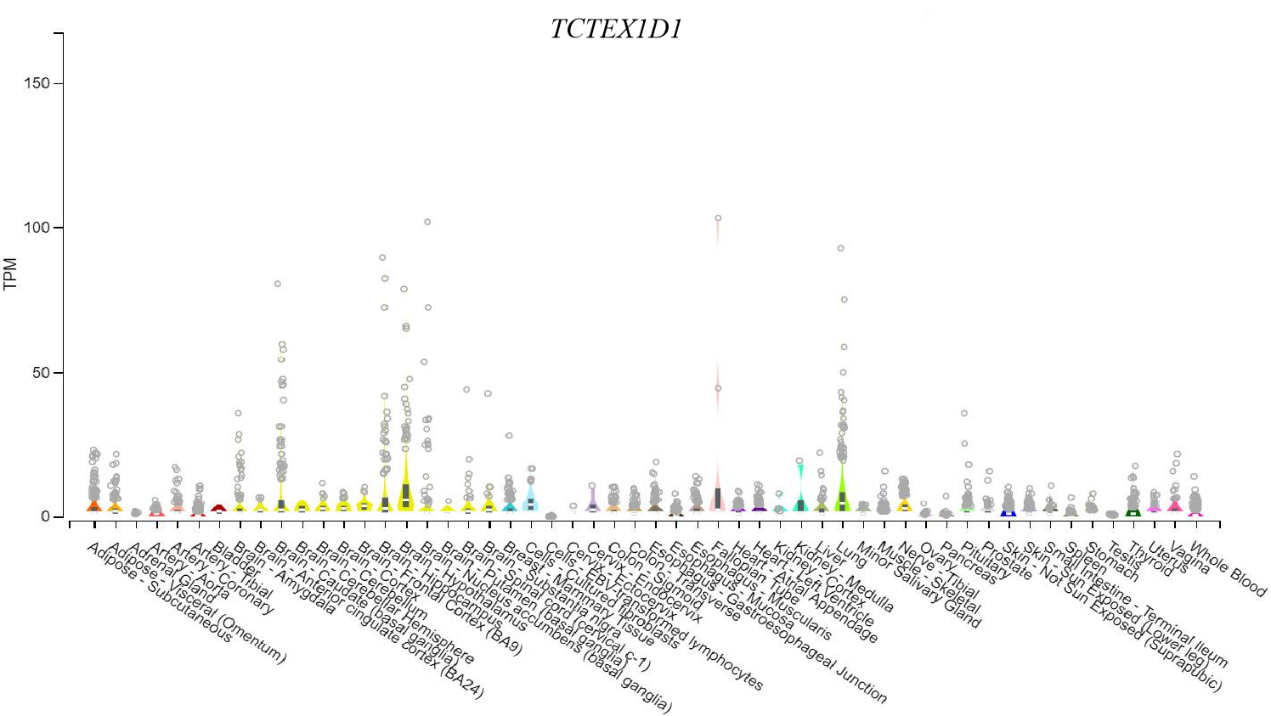


**Supplementary Figure 4. Expression of depression TWS gene *TCTEX1D1* in human tissues (data from the GTEx^[3](#_ENREF_3)^).** The x axis shows 54 tissue types. One color indicates one tissue group.


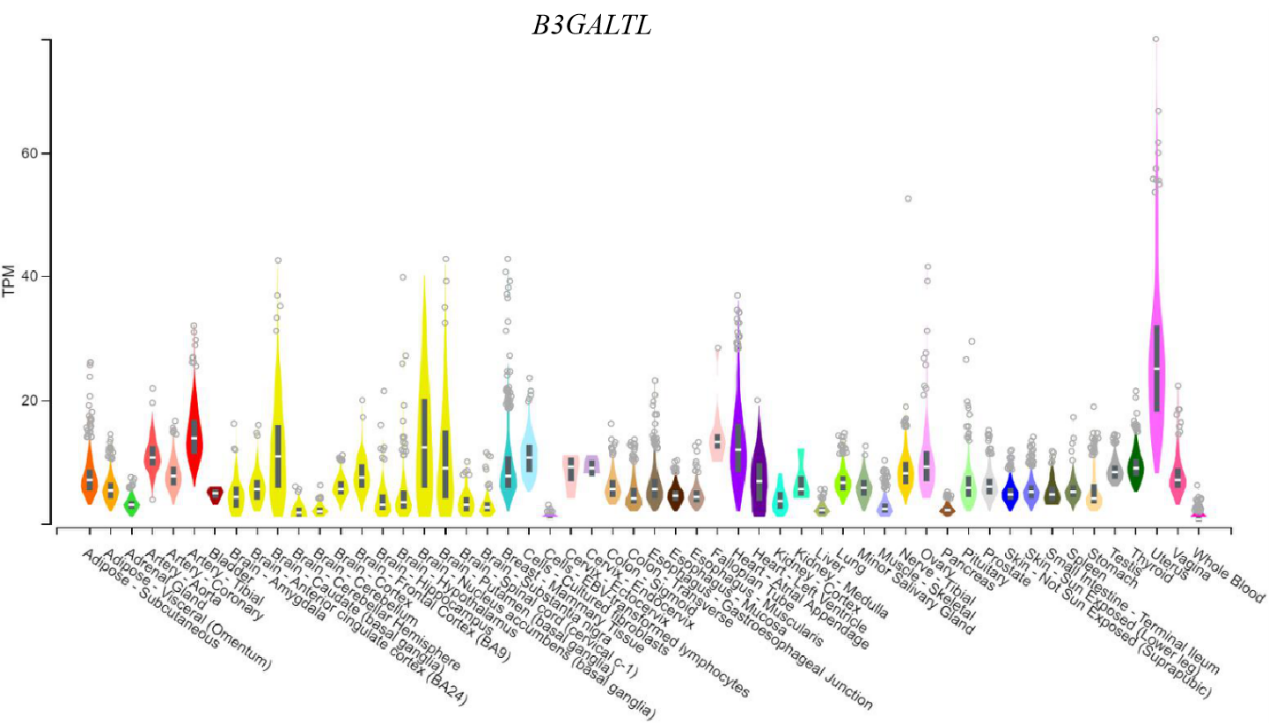


**Supplementary Figure 5. Expression of depression TWS gene *B3GALTL* in human tissues (data from the GTEx^[3](#_ENREF_3)^).** The x axis shows 54 tissue types. One color indicates one tissue group.


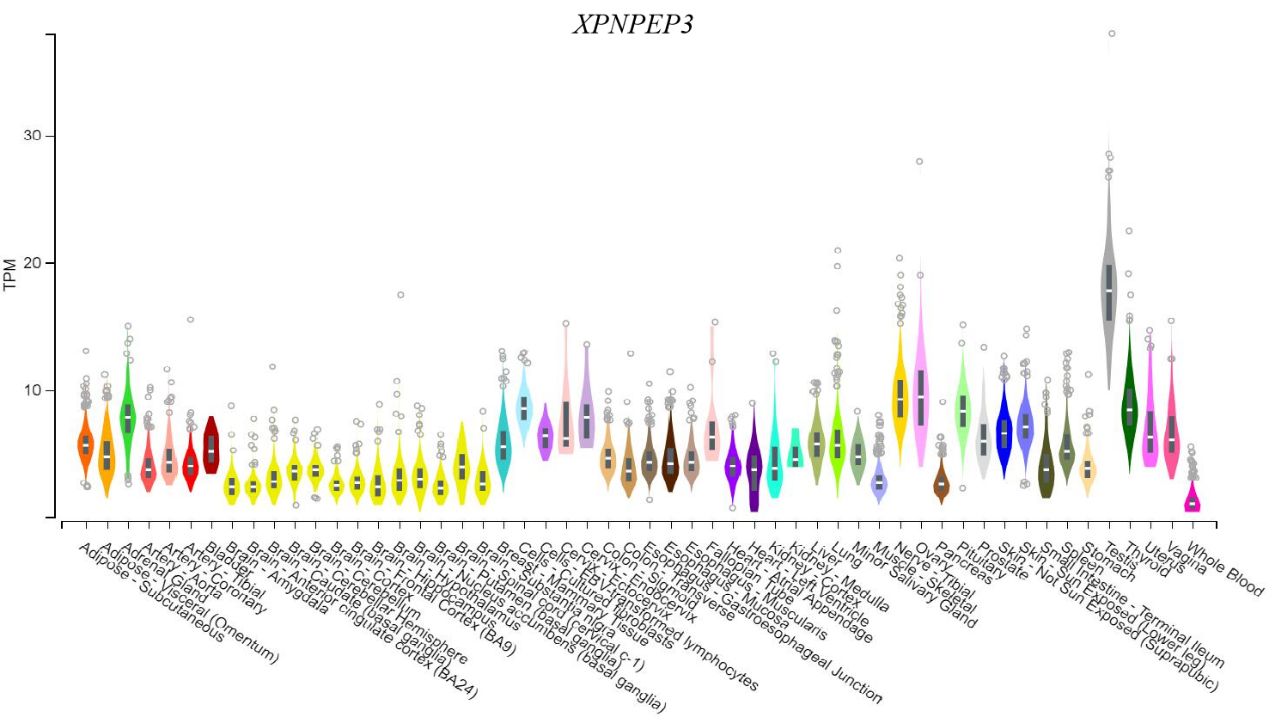


**Supplementary Figure 6. Expression of depression TWS *XPNPEP3* gene in human tissues (data from the GTEx^[3](#_ENREF_3)^).** The x axis shows 54 tissue types. One color indicates one tissue group.


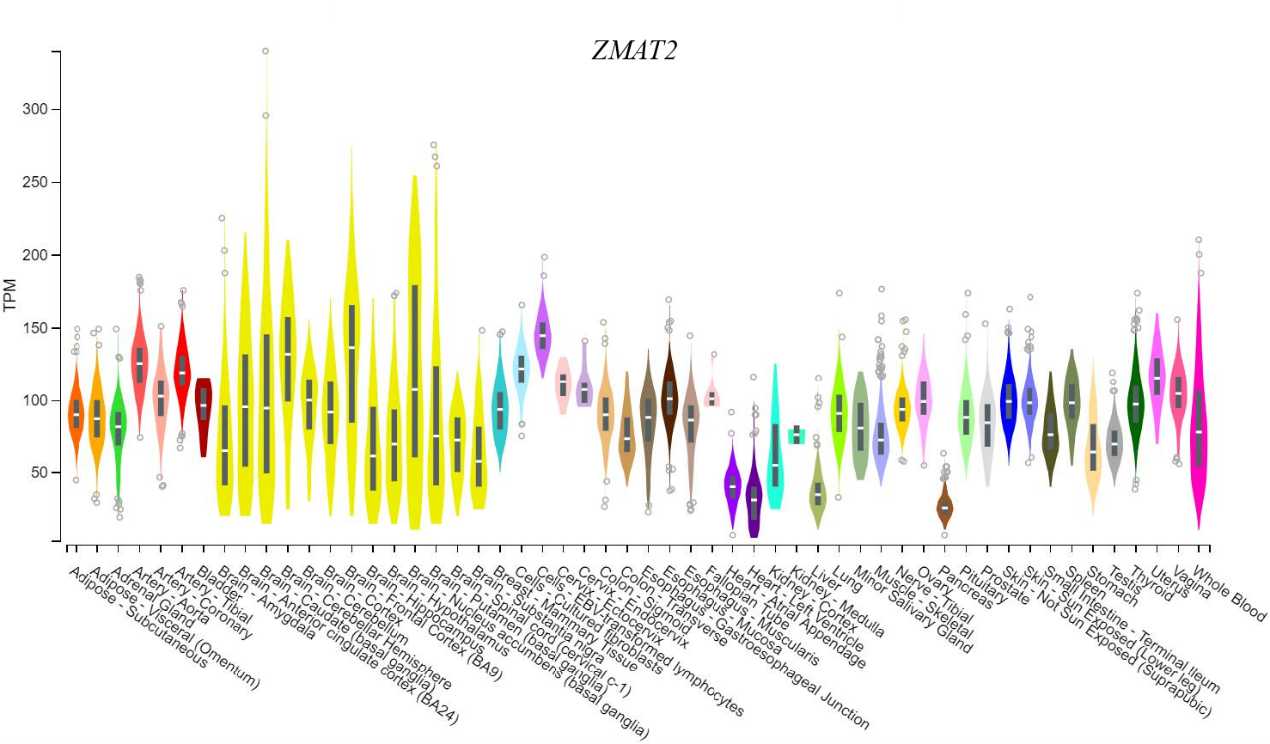


**Supplementary Figure 7. Expression of depression TWS gene *ZAMT* in human tissues (data from the GTEx^[3](#_ENREF_3)^).** The x axis shows 54 tissue types. One color indicates one tissue group.


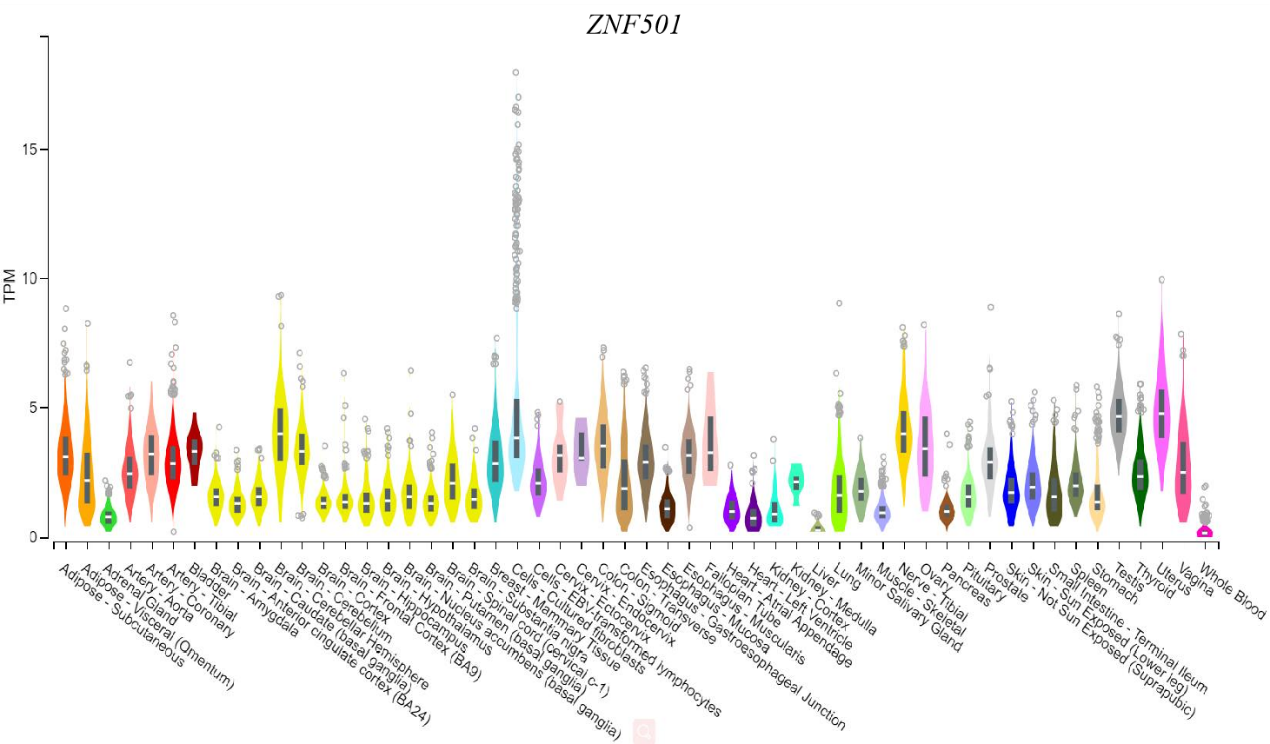


**Supplementary Figure 8. Expression of depression TWS gene *ZNF501* in human tissues (data from the GTEx^[3](#_ENREF_3)^).** The x axis shows 54 tissue types. One color indicates one tissue group.


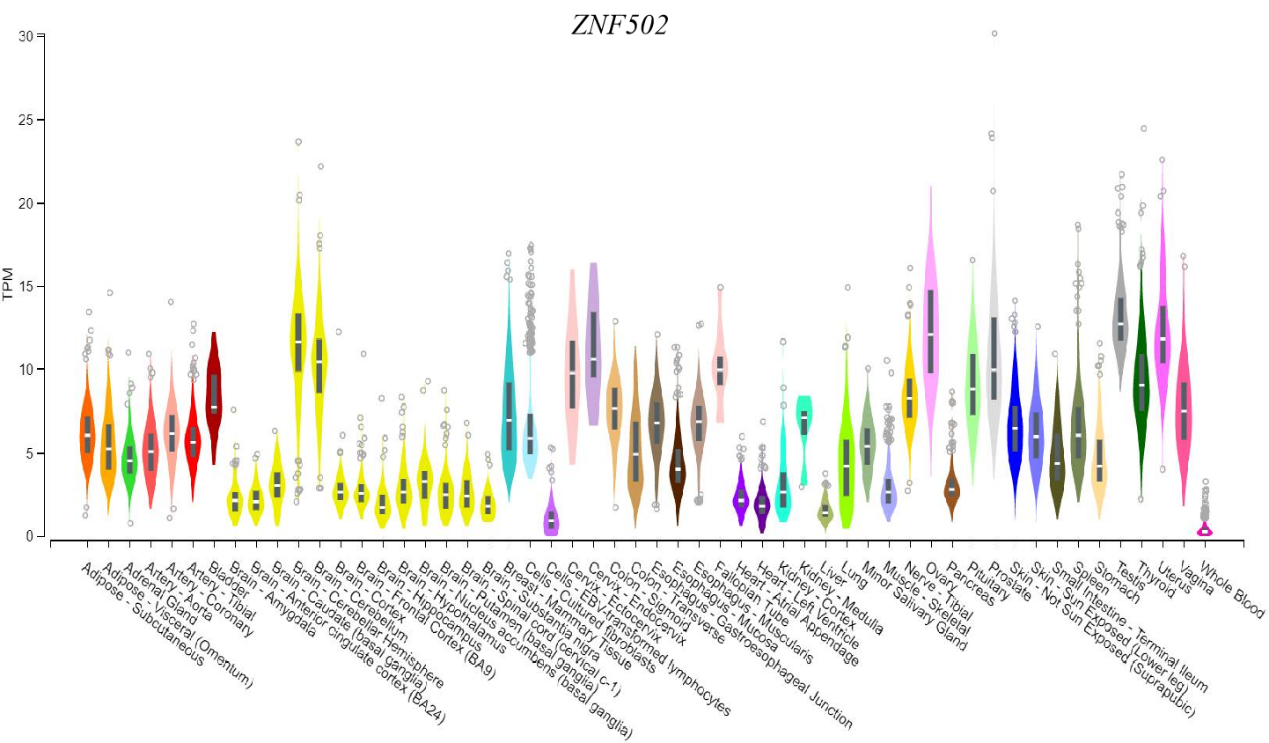


**Supplementary Figure 9. Expression of depression TWS gene *ZNF502* in human tissues (data from the GTEx^[3](#_ENREF_3)^).** The x axis shows 54 tissue types. One color indicates one tissue group.


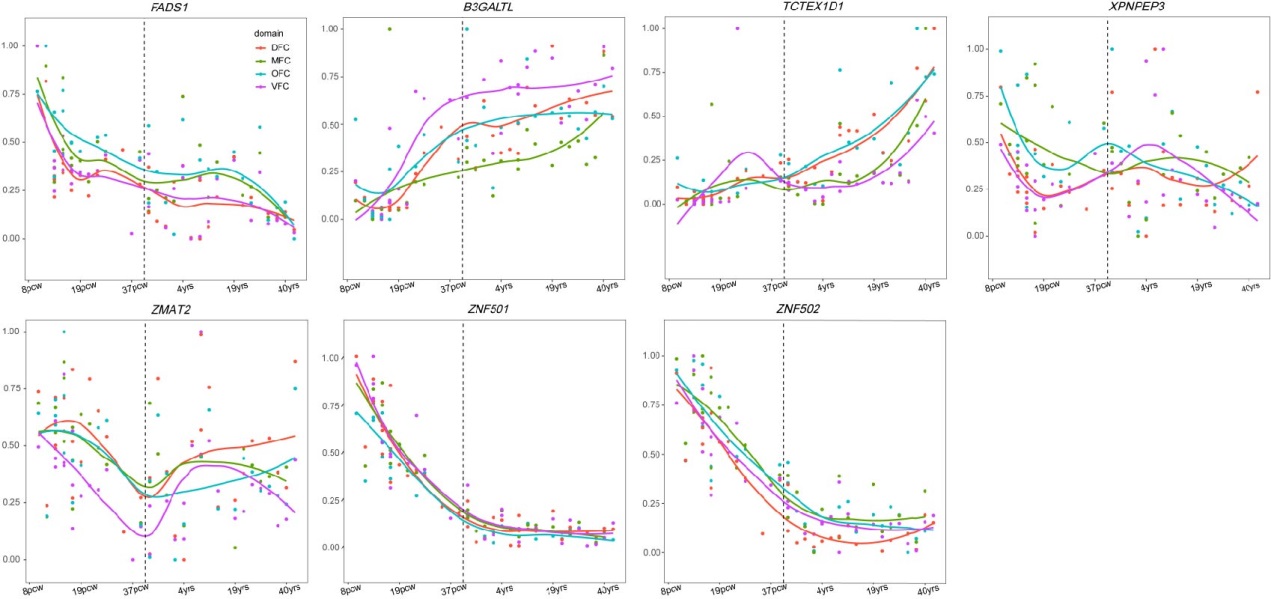


**Supplementary Figure 10. Expression patterns of the seven TWS genes in in developing and adult human brain.** The expression data from the BrainSpan[^1^](#_ENREF_1) was used for plotting.


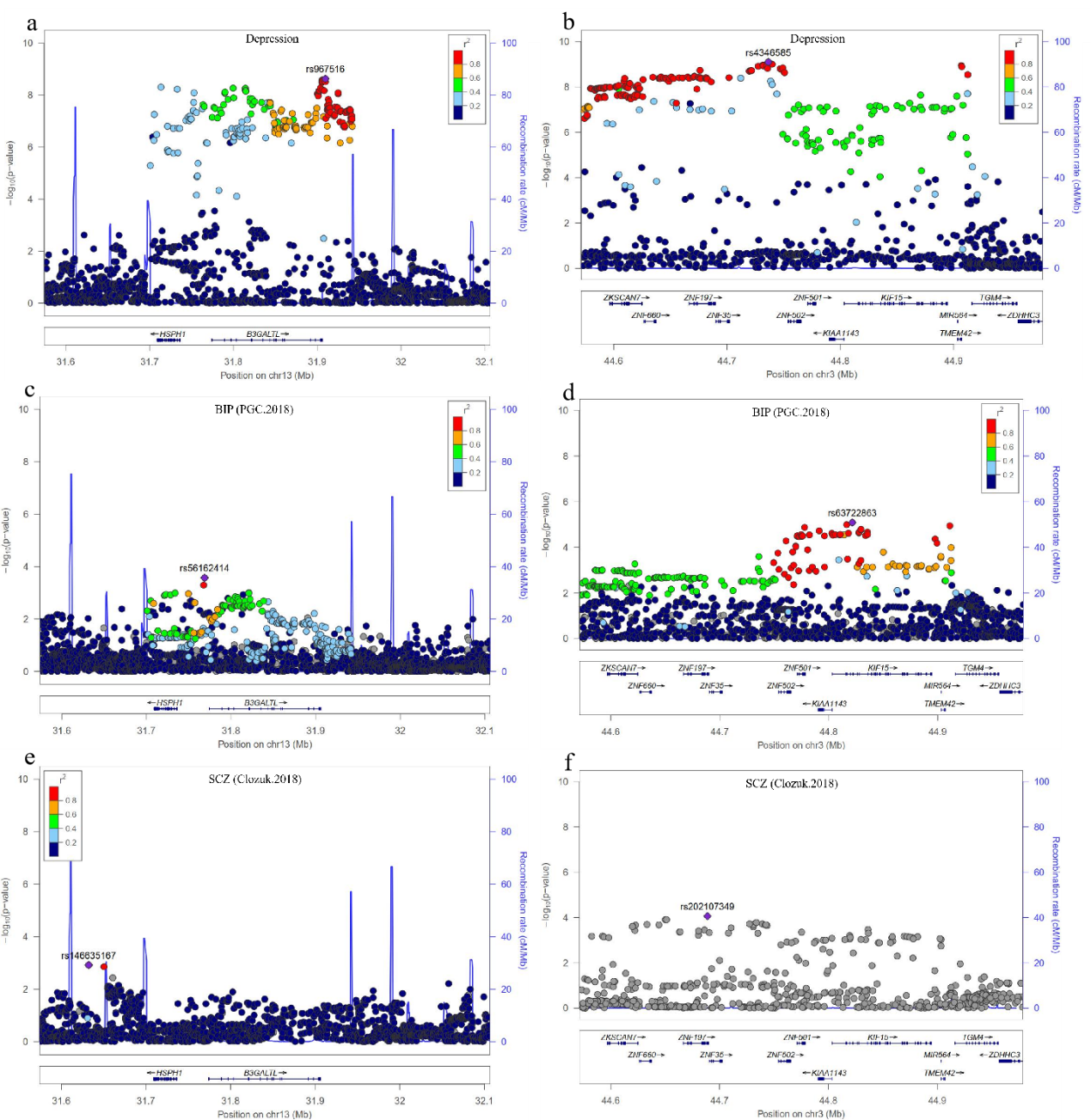


**Supplementary Figure 11. Regional association plots** **for the associations between the three TWS depression genes and depression**[**^4^**](#_ENREF_4)**, bipolar disorder**[**^5^**](#_ENREF_5) **and schizophrenia**[**^6^**](#_ENREF_6)**.**

**References**

1. Kang, H. J. et al. Spatio-temporal transcriptome of the human brain. *Nature* **478**, 483-489 (2011).

2. Skene, N. G. et al. Genetic identification of brain cell types underlying schizophrenia. *Nat Genet* **50**, 825-833 (2018).

3. Consortium, G. T. The Genotype-Tissue Expression (GTEx) project. *Nat Genet* **45**, 580-585 (2013).

4. Howard, D. M. et al. Genome-wide meta-analysis of depression identifies 102 independent variants and highlights the importance of the prefrontal brain regions. *Nat Neurosci* **22**, 343-352 (2019).

5. Stahl, E. A. et al. Genome-wide association study identifies 30 loci associated with bipolar disorder. *Nat Genet* **51**, 793-803 (2019).

6. Pardinas, A. F. et al. Common schizophrenia alleles are enriched in mutation-intolerant genes and in regions under strong background selection. *Nat Genet* **50**, 381-389 (2018).
